# Paradoxically Sparse Chemosensory Tuning in Broadly Integrating External Granule Cells in the Mouse Accessory Olfactory Bulb

**DOI:** 10.1523/JNEUROSCI.2238-19.2020

**Published:** 2020-07-01

**Authors:** Xingjian Zhang, Julian P. Meeks

**Affiliations:** ^1^University of Texas Southwestern Medical Center, Dallas, Texas 75390; ^2^University of Rochester School of Medicine and Dentistry, Rochester, NY, 14642

**Keywords:** accessory olfactory system, chemical senses, interneuron, olfaction, sensory processing, vomeronasal system

## Abstract

The accessory olfactory bulb (AOB), the first neural circuit in the mouse accessory olfactory system, is critical for interpreting social chemosignals. Despite its importance, AOB information processing is poorly understood compared with the main olfactory bulb (MOB). Here, we sought to fill gaps in the understanding of AOB interneuron function. We used 2-photon GCaMP6f Ca^2+^ imaging in an *ex vivo* preparation to study chemosensory tuning in AOB external granule cells (EGCs), interneurons hypothesized to broadly inhibit activity in excitatory mitral cells (MCs). In *ex vivo* preparations from mice of both sexes, we measured MC and EGC tuning to natural chemosignal blends and monomolecular ligands, finding that EGC tuning was sparser, not broader, than upstream MCs. Simultaneous electrophysiological recording and Ca^2+^ imaging showed no differences in GCaMP6f-to-spiking relationships in these cell types during simulated sensory stimulation, suggesting that measured EGC sparseness was not due to cell type-dependent variability in GCaMP6f performance. *Ex vivo* patch-clamp recordings revealed that EGC subthreshold responsivity was far broader than indicated by GCaMP6f Ca^2+^ imaging, and that monomolecular ligands rarely elicited EGC spiking. These results indicate that EGCs are selectively engaged by chemosensory blends, suggesting different roles for EGCs than analogous interneurons in the MOB.

**SIGNIFICANCE STATEMENT** The mouse accessory olfactory system (AOS) interprets social chemosignals, but we poorly understand AOS information processing. Here, we investigate the functional properties of external granule cells (EGCs), a major class of interneurons in the accessory olfactory bulb (AOB). We hypothesized that EGCs, which are densely innervated by excitatory mitral cells (MCs), would show broad chemosensory tuning, suggesting a role in divisive normalization. Using *ex vivo* GCaMP6f imaging, we found that EGCs were instead more sparsely tuned than MCs. This was not due to weaker GCaMP6f signaling in EGCs than in MCs. Instead, we found that many MC-activating chemosignals caused only subthreshold EGC responses. This indicates a different role for AOB EGCs compared with analogous cells in the main olfactory bulb.

## Introduction

Social behavior involves multimodal sensory inputs, information processing cascades conducted by multilevel neural circuits, and complicated behavioral outputs. Within each brain region, interactions between principal neurons and interneurons form neural circuit motifs, the fundamental computational building blocks for information processing (Braganza and Beck, 2018). In mice and many mammals, the accessory olfactory system (AOS) is required for the expression of typical social behaviors, but many fundamentals of its organization and function remain unclear. The AOS detects nonvolatile chemosignals including pheromones (for intraspecies communication) and kairomones (for interspecies communication; Mohrhardt et al., 2018). In rodents, the first dedicated neural circuit for chemosignal information processing is the accessory olfactory bulb (AOB).

AOB principal neurons, known as mitral cells (MCs), are tightly modulated by local GABAergic interneurons and GABAergic modulation in the AOB has significant behavioral impacts (Kaba and Nakanishi, 1995; Brennan, 2001; Hendrickson et al., 2008). For example, microinfusion of GABAergic transmission blocker to AOB can block pregnancy (Kaba and Keverne, 1988). Many studies have implicated AOB GABAergic interneurons in social behaviors and experience-dependent chemosensory plasticity (Brennan et al., 1995; Matsuoka et al., 1997, 2004; Brennan and Binns, 2005; Kaba and Huang, 2005; Araneda and Firestein, 2006; Hendrickson et al., 2008; Cansler et al., 2017; Gao et al., 2017). Cumulatively, these observations suggest that MC–interneuron communication in the AOB is critical for rodent physiology and behavior.

Despite recent progress, we lack information about how MCs interact with several inhibitory neural types. The AOB has a variety of interneurons that were classified based on their location and morphology (Larriva-Sahd, 2008; Moriya-Ito et al., 2013). Juxtaglomerular cells (JGCs) are found within the superficial glomerular layer, where excitatory sensory inputs from the vomeronasal organ (VNO) enter the AOB. Internal granule cells (IGCs), the most abundant and well studied AOB interneuron type, are located in the internal granule layer. External granule cells (EGCs) are found in the external cell layer alongside the somas of MCs. JGCs, similar to counterparts in the main olfactory bulb (MOB), are thought to modulate the input to the MCs via their synaptic connection with VNO axon terminals and MC apical dendrites within glomeruli (Geramita and Urban, 2017). EGCs and IGCs, in contrast, are thought to modulate MC activity via deeper reciprocal dendrodendritic connections (Taniguchi and Kaba, 2001; Castro et al., 2007). Relative to JGCs and IGCs, there is very little information about the physiology or function of AOB EGCs; just one targeted study of their intrinsic features has been reported (Maksimova et al., 2019).

Cells that appear analogous to AOB EGCs have been studied in the MOB. Specifically, MOB parvalbumin-expressing interneurons in the external plexiform layer (PV-EPL interneurons) resemble EGCs in their morphologies and apparent broad connectivity with MCs. These MOB PV-EPL interneurons were shown to integrate excitatory information from multiple MCs, resulting in broader odorant receptive fields than MCs (Kato et al., 2013). In this context, the broad tuning of PV-EPL interneurons, along with their capacity for lateral inhibition of MCs, supported so-called divisive normalization or gain scaling. This divisive normalization function resulted in increased levels of MC inhibition as more MCs were recruited by the sensory stimulus, an effect that is thought to limit population activity and preserve sensory discrimination across concentration ranges (Kato et al., 2013; Miyamichi et al., 2013). Given the similarities between MOB PV-EPL interneurons and AOB EGCs, we hypothesized that AOB EGCs would display broad chemosensory tuning and carry out divisive normalization of MCs through their inhibitory dendrodendritic synapses.

Here, we describe the first targeted investigation of AOB EGCs in the context of chemosensory function. Using a Cre-expressing transgenic mouse (*Cort*-T2A-Cre), which selectively labels subsets of AOB EGCs (Taniguchi et al., 2011; Maksimova et al., 2019), we measured EGC activation by AOS ligands in an *ex vivo* preparation of the early AOS, finding unexpectedly sparse activation compared with MCs and JGCs. Loose-seal cell-attached recordings on EGCs indicated no differences in GCaMP6f performance in EGCs compared with MCs in the context of naturalistic stimulation. Whole-cell *ex vivo* recordings revealed broad subthreshold activation of EGCs, but most EGCs only spiked in response to natural chemosensory blends (e.g., mouse urine or feces). The lack of broad spiking activity in AOB EGCs suggests that EGCs do not support divisive normalization in the context of small numbers of odorants, but may do so in rich pheromone environments. Overall, these studies provide new information about the role of AOB EGCs in AOS sensory processing and place important constraints on our models of AOB circuit function.

## Materials and Methods

### 

#### Mice

All animal procedures were in compliance with the UT Southwestern Institutional Care and Use Committee. Mice used in this research were C57BL/6J, unless otherwise noted. *Cort*-T2A-Cre and *Gad*-IRES-Cre (Taniguchi et al., 2011) were from The Jackson Laboratory (stock #010910 and #028867). *Pcdh21*-Cre (Nagai et al., 2005) mice were shared by the laboratory of Timothy Holy (Washington University School of Medicine, Saint Louis, MO) with permission from the originating institution. Both male and female mice were used in all experiments, and the results were pooled. Twenty-seven mice (14 females and 13 males) were used for EGC Ca^2+^ imaging. Five mice (three females and two males) were used for JGC Ca^2+^ imaging. Nine mice (four females and five males) were used for MC Ca^2+^ imaging. For slice electrophysiological experiments, 20 mice (17 females and 3 males) were used for EGC patch-clamp recordings and cell-attached recordings; 10 mice (three females, and 7 males) were used for MC patch-clamp recordings and cell-attached recordings. For EGC *ex vivo* patch-clamp recording, 16 mice (10 females and 6 males) were used.

#### Stimuli and reagents

Female mouse fecal extracts and urine were prepared as previously described (Nodari et al., 2008; Meeks et al., 2010; Doyle et al., 2016). Fecal extracts and urine were pooled across subjects of the same sex, strain, and age, then aliquoted and stored at −80°C. Just before each experiment, aliquots were thawed and diluted in control Ringer's saline solution containing the following (in mm): 115 NaCl, 5 KCl, 2 CaCl_2_, 2 MgCl_2_, 25 NaHCO_3_, 10 HEPES, and 10 glucose. For VNO stimulation, the fecal extracts were diluted at 1:300, and the urine was diluted at 1:100, concentrations that activate approximately equal numbers of AOB MCs in the *ex vivo* preparation (Doyle et al., 2014).

All sulfated steroids were purchased from Steraloids. The sulfated steroid panel includes A7864 (5-androsten-3β, 17β-diol disulfate, disodium salt), A6940 (4-androsten-17α-ol-3-one sulfate sodium salt), A7010 (4-androsten-17β-ol-3-one sulfate, sodium salt), E0893 [1, 3, 5(10)-estratrien-3, 17α-diol 3-sulfate, sodium salt], E1050 [1, 3, 5(10)-estratrien-3, 17β-diol disulfate, disodium salt], E4105 (4-estren-17β-ol-3-one sulfate, sodium salt), P3817 (5α-pregnan-3α-ol-20-one sulfate sodium salt), P3865 (5α-pregnan-3β-ol-20-one sulfate, sodium salt), P8168 (5β-pregnan-3α-ol-20-one sulfate, sodium salt), Q1570 (4-pregnen-11β, 21-diol-3, 20-dione 21-sulfate, sodium salt), and Q3910 (4-pregnen-11β, 17, 21-triol-3, 20-dione 21-sulfate, sodium salt). Twenty millimolar stock solutions of A7864, EML, and Q1570 were prepared in H_2_O, the 20 mm stock solution of all other sulfated steroids were prepared in methanol. Upon use, stock solutions were diluted at 1:2000 into the Ringer's solution (10 μm working concentration). Methanol was diluted at 1:2000 into the Ringer's solution as a vehicle control.

#### Targeted GCaMP6f expression

GCaMP6f expression in AOB neurons was achieved by injecting AAV.CAG.Flex.GCaMP6f.WPRE.SV40 (Chen et al., 2013) to the corresponding Cre mouse lines. To achieve optimal GCaMP6f expression, different adeno-associated virus (AAV) pseudotypes were used. AAV9 (catalog #AV9-PV2816, Penn Vector Core) was used on *Cort*-T2A-Cre for EGC labeling, and *Gad2*-IRES-Cre (Taniguchi et al., 2011) for JGC labeling. AAV5 (catalog #AV5-PV2816, Penn Vector Core) was used on *Pcdh21*-Cre for MC labeling, confirming the efficacy of this AAV pseudotype for MCs (Rothermel et al., 2013).

Adult mice 8–12 weeks of age were used for virus injection. Intracranial injections were performed on a customized stereotaxic device that rotated the mouse head such that the rostral end of the head tilted up ∼30°. Mice were anesthetized via isoflurane inhalation using a SomnoSuite Small Animal Anesthesia System (Kent Scientific). For each animal, ∼180 to 300 nl of viral vector (≥1e^13^ μg/ml) was injected into the AOB. The bilateral coordinates, measured from Bregma, were lateral approximately +1000 μm and anterior approximately +4150 μm for 8-week-old adult mice. Depth coordinate was ∼3300 μm beneath the skull surface. After virus injection, the animals were allowed to recover for at least 3 weeks before being used for experiments.

Additionally, the *Cort*-T2A-Cre mouse line crossed with Ai148D mice (TIT2L-GC6f-ICL-tTA2)-D (stock #030328, The Jackson Laboratory) to transgenically express GCaMP6f in EGCs. Eight of these animals were used in *ex vivo* imaging, producing 10 EGC data instances. We observed no discernible differences between the *ex vivo* GCaMP6f imaging results of Ai148D animals and virally driven animals (data not shown), so these instances are compiled in one dataset and analyzed together.

#### VNO-AOB *ex vivo* preparation

*Ex vivo* preparations were performed as described previously (Meeks and Holy, 2009; Doyle et al., 2014). Briefly, mice were anesthetized by isoflurane inhalation, followed by rapid decapitation into the ice-cold artificial CSF (aCSF). After removing the scalp, the snout and olfactory bulbs were separated from the rest of the skull, and the snout was then halved along the midline, maintaining the VNO AOB from the right hemisphere. The resulting tissue was affixed to a plastic plank with tissue adhesive (Krazy Glue, Elmer's Products) and placed into a custom perfusion chamber where secondary dissections were performed. In this chamber, room temperature (22–25°C) oxygenated aCSF was rapidly superfused over the tissue at a rate of 5–8 ml/min. aCSF contained the following (in mm): 125 NaCl, 2.5 KCl, 2 CaCl_2_, 1 MgCl_2_, 25 NaHCO_3_, 1.25 NaH_2_PO_4_, 25 glucose, 3 *myo*-inositol, 2 sodium pyruvate, and 0.4 sodium ascorbate. The septal cartilage was carefully removed, exposing the septal tissue containing the axons from VNO to the oxygenated aCSF. The sample was then transferred to a second, custom-built tissue chamber with a rotatable platform. A small cut was made at the anterior end of the VNO capsule, through which polyimide tubing (inner diameter, 0.0045 inch; wall, 0.00050 inch; A-M Systems) was inserted for stimulus delivery. Stimulation solution was pressurized at 9–12 psi giving an effective flow rate of 0.2–1 ml/min. Valve opening was controlled by an Automate Scientific perfusion system with a ValveLink8.2 controller. Once cannulated, the platform was rotated so that the AOB facing upward to facilitate 2-photon imaging on an upright microscope.

#### Acute slice preparation

Mice were anesthetized with isoflurane and immediately decapitated into ice-cold oxygenated aCSF with an additional 9 mm MgCl_2_. Brains were then extracted, and a vertical cut at the prefrontal cortex was made and the anterior part containing the olfactory bulbs was preserved. Another vertical cut along the midline separated the two hemispheres, and both were embedded in aCSF containing 3% low-melt agarose at 37°C. The agarose block was then mounted on an angled slicing platform on a vibrating microtome (VT1200, Leica). The slicing blade ran at an angle of ∼12° off-sagittal, running from caudal/medial to rostral/lateral. The slices were then collected in a recovery chamber containing oxygenated room temperature aCSF with 0.5 mm kynurenic acid. Slices were allowed to recover for at least 30 min before being used for patch-clamp recording.

#### Experimental design and statistical analysis

##### *Ex vivo* 2-photon GCaMP6f imaging.

Adult mice 11–16 weeks of age were used for imaging. *Ex vivo* preparations in the customized chamber were placed into a custom adapter on a Thorlabs Acerra upright 2-photon microscope system equipped with an XLUMPlanFLN 20× objective (Olympus) and a fast-scanning resonant galvanometer along one of the two principal axes. To excite GCaMP6f fluorescence, 910 nm light (average power measured at the laser, 2100 mW; power transmission for imaging, 25–35%) was used. Images with pixel dimensions 512 × 512 were acquired at 30 frames per second (fps) and synchronized with a stimulus delivery system (Automate Scientific) via Axon Clampex 10 software (Molecular Devices). Episodic stimulation sessions, consisting of 1 s prestimulation VNO Ringer's solution flush, 8 s of VNO stimuli, and 11 s poststimulation VNO Ringer's solution flush, were used to present multiple repeats per cell. Across sessions, stimulus presentation order was randomized to reduce the impact of potential stimulus order effects.

##### Acute slice whole-cell patch-clamp electrophysiology.

Adult mice 11–16 weeks of age were used for acute slice electrophysiology experiments. Acute slice electrophysiology was performed on the same upright 2-photon microscope used in *ex vivo* imaging. Slices were placed in a tissue chamber (Warner Instruments) and warmed to 28–30°C by a temperature controller (Warner Instruments). GCaMP6f-expressing neurons were identified using the same laser setup with *ex vivo* imaging. Thin borosilicate glass electrodes (TW150, World Precision Instruments) were pulled using a horizontal puller (P1000, Sutter Instruments). The electrodes were then filled with standard internal solution containing 115 mm K-gluconate, 20 mm KCl, 10 mm HEPES, 2 mm EGTA, 2 mm MgATP, 0.3 mm Na_2_GTP, and 10 mm Na phosphocreatine at pH 7.37. Alexa Fluor 568 (166 μm; Thermo Fisher Scientific) was added for visualization under a 2-photon microscope. Pipette resistance ranged from 7 to 10 MΩ for EGC patch clamp, and 6–8 MΩ for MC patch clamp. The electrodes were controlled with a MicroStar motorized micromanipulator (Scientifica). In the experiments probing the relationship between GCaMP6f signal and action potentials, after whole-cell configuration was formed, cells went through the voltage-clamp and current-clamp protocols. Our voltage-clamp protocol started at −70 mV, followed by a 10 s train of 20 Hz depolarization pulses to 0 mV (2 ms pulse width). Under the current-clamp mode, we inject either sustained step currents or 2 ms current pulse trains to evoke action potentials at desired frequencies. GCaMP6f signals were recorded simultaneously using the same imaging parameters as *ex vivo* Ca^2+^ imaging experiments. The GCaMP6f signal was then extracted using custom MATLAB programs.

##### Acute slice loose-seal cell-attached recording and local field stimulation.

We used the same acute brain slice preparation for loose-seal cell-attached recordings as for whole-cell patch-clamp recordings. Pulse stimulation of the glomerular layer was applied using a stimulus isolator (model A365, World Precision Instruments) at 20 Hz for 10 s (5 ms pulse width). Stimulation was achieved via an ACSF-filled theta-glass electrode with a ∼30 µm tip. Spontaneous and evoked action potentials were recorded using AaCSF-filled borosilicate glass pipettes (resistance, 6–8 MΩ) in the loose-seal configuration.

##### *Ex vivo* whole-cell patch-clamp electrophysiology.

The *ex vivo* preparation setup for whole-cell patch-clamp experiments was the same as for the *ex vivo* imaging. *Cort*-T2A-Cre mice were crossed with Cre-dependent tdTomato effector mice (“Ai9” mouse line; stock #007909, The Jackson Laboratory) to label Cort^+^ cells. Adult offspring mice 11–16 weeks of age were used. Alexa Fluor 488 (100 μm; Thermo Fisher Scientific) was added in the standard internal solution for electrode visualization under a 2-photon microscope. Target cells were identified and approached using the “approach” mode of the micromanipulator (to facilitate penetrating the tissue without tearing the glomerular layer) before achieving the whole-cell configuration. After the whole-cell configuration was achieved, cells were held in current-clamp mode. Upon break-in, the resting membrane potential of the cell was measured, and a steady-state holding current was applied throughout the experiment to maintain the initial resting membrane potential. The same panel of monomolecular ligands and natural stimuli were applied to the VNO as the for *ex vivo* Ca^2+^ imaging experiments.

All recordings were amplified via a MultiClamp 700B amplifier (Molecular Devices) at 20 kHz and were digitized by a DigiData 1440 analog–digital converter via pClamp 10.5 software (Molecular Devices; RRID:SCR_011323). Data were analyzed by custom software written in MATLAB, and graphs were created using MATLAB and R (ggplot2).

##### *Ex vivo* 2-photon GCaMP6f imaging analysis.

Raw 2-photon Ca^2+^ imaging analysis was performed using customized MATLAB scripts. Regions of interest (ROIs) were manually selected and change in fluorescence (ΔF/F) values were extracted by comparing the change in fluorescence during stimulation to 30 frames (∼1 s) before each stimulus session. Because EGCs were relatively rare in the field of view, and very dim at rest, before each experiment we manually pulsed the VNO with each of the stimuli in the panel, which revealed stimulus-responsive cells in the field of view. Throughout this study, only cells (MCs, EGCs, and JGCs) that responded to at least one stimulus were included in the analysis. Further analysis of ΔF/F signals was performed using customized R scripts, and graphs were made using ggplot2. ΔF/F responses were averaged over four or more trials for each stimulus. Because the latency to peak for each cell and each specific preparation can vary (typically between 7 and 12 s from stimulation onset), we used average response curves to determine a continuous series of samples during which the ΔF/F value is >50% of the peak value. For each individual repeat, we integrated the ΔF/F intensity during this time window. We assessed the statistical reliability of the stimulus responsiveness of each cell using the unpaired Student's *t* test, comparing each stimulus to the vehicle control trials. We considered a cell to be responsive to a stimulus if the *p* value compared with negative control trials was <0.05 and peak ΔF/F was >0.1. These criteria were met by most cellular responses, but we noticed several cell–stimulus pairs with responses that greatly surpassed the 0.1 ΔF/F threshold, but demonstrated *p* values between 0.05 and 0. 1 due to the relatively small repeat number (≥3). To avoid the possibility of falsely rejecting these strong responses, we added a second statistical criterion with an elevated 0.3 ΔF/F amplitude threshold and a slightly relaxed statistical threshold of *p* < 0.1. For heat map displays throughout the article (see Figs. 2*B*, 3*B*, 4*D*), average peak ΔF/F value was used to represent the response strength of each cell toward to each stimulation. The cumulative distribution of EGC, JGC, and MC tuning was evaluated using a Kolmogorov–Smirnov test (see Figs. 5*A*, 5*B*, 7*C*). Heat map displays of chemosensory tuning (see Figs. 2*B*, 3*C*, 4*D*) were manually arranged based on the ligand responsivity of each cell.

##### Simultaneous GCaMP6f imaging and acute slice electrophysiology analysis.

For analysis of current-clamp stimulation experiments, we measured the peak ΔF/F following each spike, and ΔF/F-to-spike relationships were analyzed by two-way ANOVA, (see Fig. 6*D*). For loose-seal cell-attached recordings (see Fig. 6*E–I*), electrical stimulation was used to stimulate the glomerular layer, resulting in variable spike timing in downstream neurons (i.e., total number of spikes and interspike interval distributions varied). For comparisons of the ΔF/F–spiking relationship in these experiments, we analyzed the first 20 consecutive spikes within the ΔF/F rising phase (i.e., before the overall peak/plateau). If a cell fired <20 spikes in a given trial, we analyzed the bout with the largest number of spikes. The average interspike intervals were 0.16 ± 0.01 s for MCs and 0.26 ± 0.09 s for EGCs. To assess the effect of the initial action potentials on the GCaMP6f signal, we calculated the ΔF/F of a fixed time window (3 frames or 10 frames; 30 fps) immediately after the first evoked spike onset. The amplitude of ΔF/F over these frames was plotted against the number of spikes that occurred within that time window (see Fig. 6*H*), and compared using two-way ANOVA. We generated spike-to-ΔF/F relationship of the first 20 evoked action potentials by measuring the peak ΔF/F immediately following each spike and evaluated using a two-way ANOVA (see Fig. 6*I*, left). The average number of spikes to reach the 0.1 and 0.3 ΔF/F threshold was compared using Student's *t* test (see Fig. 6*I*, right).

##### Two-photon *ex vivo* whole-cell patch-clamp electrophysiology analysis.

In these experiments, each round of stimulation included 1 s of prestimulation flush, 8 s of stimulation, and 11 s of poststimulation flush. The stimulation panel was split into two bouts, each consisting of six sulfated steroids, two naturalistic stimuli, and the vehicle control, delivered in randomized orders. Two bouts were used to cover the entire stimulation panel, and at least three full repeats of the full stimulus panel were used for all analyzed experiments. The membrane voltage was recorded at 20 kHz during the stimulation administration, and for all comparisons except spike analysis, the data were downsampled by decimation by a factor of 100. For each repeat, we calculated the average value of the top 5% of voltage reads in a static time window between 1.5 and 10 s following the stimulus onset and used the value to quantify the subthreshold activity. The response to each stimulus was compared with the vehicle control using the Wilcoxon rank sum test (see Fig. 7*B*; three or more repeats per stimulus). All stimulus responses with *p* < 0.05 were considered effective. Comparisons of EGC and MC tuning distribution revealed by GCaMP6f imaging and EGC responsiveness revealed by *ex vivo* whole-cell patch clamp were conducted using the Kolmogorov–Smirnov test (see Fig. 7*D*).

## Results

### Implementation of cell type-specific GCaMP6f Ca^2+^ imaging in the AOB *ex vivo*

**Figure 1.**
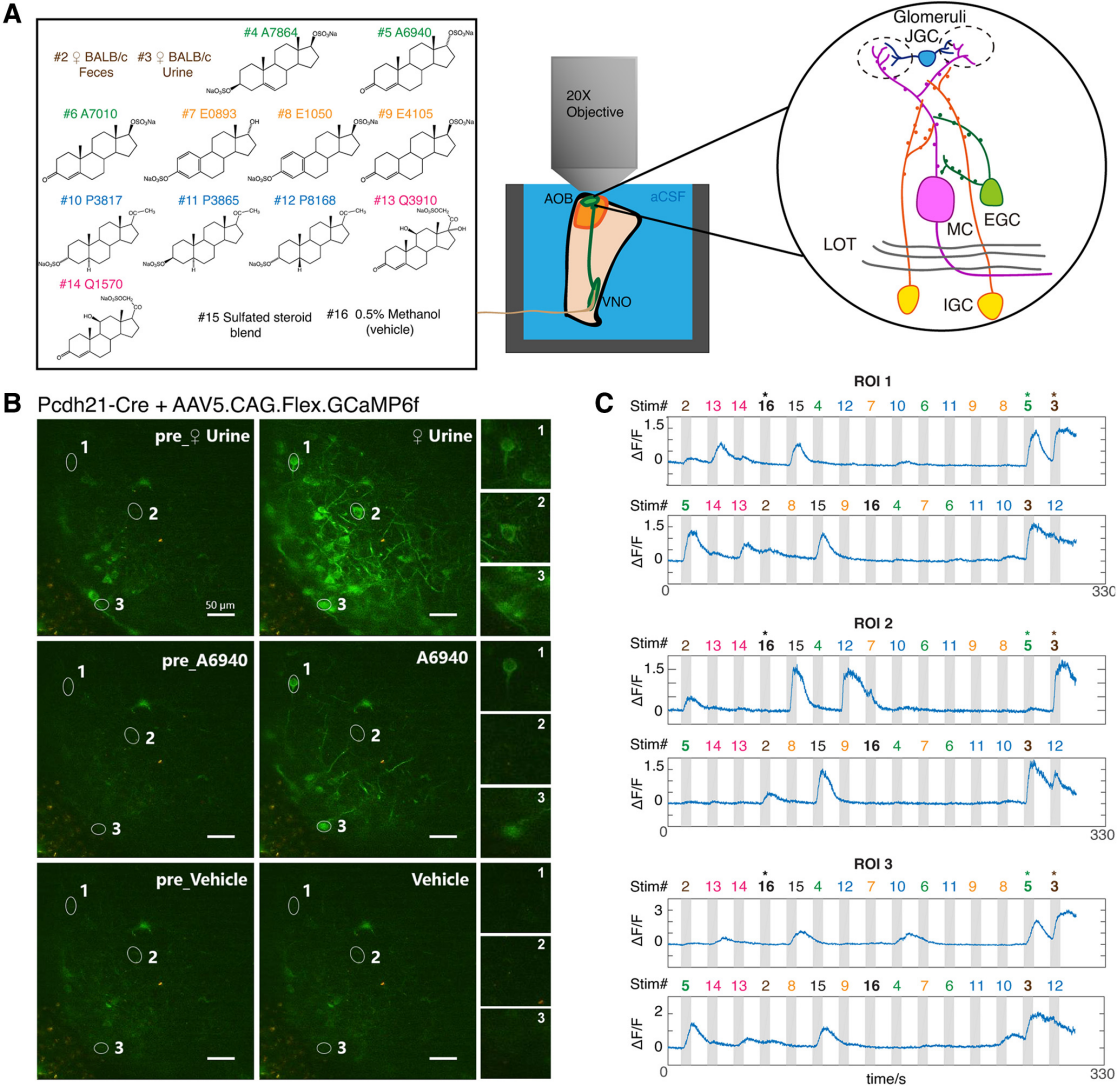
***A***, Overview of e*x vivo* Ca^2+^ imaging. Left, The stimulus panel delivered to the VNO to drive activity in the AOB. Included are natural ligand blends (1:100 diluted BALB/c mouse urine and 1:300 diluted mouse feces) and 11 monomolecular sulfated steroids at 10 μm. Right, Diagram of AOB circuit. ***B***, Raw images of GCaMP6f fluorescence during *ex vivo* Ca^2+^ imaging experiments on AOB MCs. MCs expressed GCaMP6f via the infusion of Cre-dependent AAVs into the AOBs of *Pcdh21*-Cre transgenic mice ≥3 weeks before the recordings. Numbered regions of interest denote three highlighted MCs with different tuning preferences. Scale bar, 50 μm. ***C***, ΔF/F measurements from the three cells highlighted in ***B*** across two randomized repeats. Numbers above the gray vertical bars indicate the stimulus being applied, with colors matched to the stimulus panel in ***A***.

The mouse AOB remains one of the most poorly understood principal sensory circuits in the mammalian brain. A large reason for this deficiency is the limited number of studies on the sensory responses of AOB neurons. Several *in vivo* and *ex vivo* studies have investigated MC sensory responses, but studies of interneuron function are severely lacking (Luo et al., 2003; Hendrickson et al., 2008; Ben-Shaul et al., 2010; Meeks et al., 2010; Doyle et al., 2016). We used combined *ex vivo* sensory preparations that retain VNO–AOB connectivity (Meeks and Holy, 2009) with 2-photon GCaMP6f imaging to measure Ca^2+^ signals in specific AOB neuronal populations (Fig. 1*A*).

An important consideration for any study of chemosensory tuning is that measured receptive fields critically depend on the choice of chemosensory cues and concentrations. Some physiological studies of AOB tuning have exclusively used natural blends of chemosensory cues (e.g., dilute urine and saliva; Hendrickson et al., 2008; Ben-Shaul et al., 2010; Tolokh et al., 2013), whereas others have used both of natural chemosignal blends and monomolecular VNO ligands (Meeks et al., 2010; Doyle et al., 2016; Doyle and Meeks, 2017). We chose to use both natural and monomolecular stimuli; we selected a panel that included diluted mouse urine, fecal extracts, and monomolecular sulfated steroid ligands similar to those used by previous studies (Meeks et al., 2010; Turaga and Holy, 2012; Doyle et al., 2016). We first recorded sensory tuning to this panel of odorants in AOB MCs by virally or transgenically driving GCaMP6f in *Pcdh21*-cre transgenic mice (Nagai et al., 2005). We observed reliable, time-locked, stimulus-driven chemosensory activity in populations of AOB MCs across multiple stimulus trials (Fig. 1*B*,*C*, Movie 1). GCaMP6f responses to 8 s stimulus trials were large in amplitude (ΔF/F peak amplitudes, ∼0.4 to ∼3.2) and slow to peak and decay (peak time, 7–12 s from stimulation onset; decay time, 8–14 s), which is consistent with the time course of action potential firing observed in MCs with similar stimulation conditions (Hendrickson et al., 2008; Meeks and Holy, 2009). The establishment of GCaMP6f 2-photon Ca^2+^ imaging in the *ex vivo* preparation allowed us to investigate sensory tuning properties of genetically defined AOB cell types.

Movie 1. An ex *vivo* 2-photon GCaMP6f Ca^2+^ imaging video 18X speed) showing Pcdh21+ MCs activated by VNO chemosensory stimulation. 10.1523/JNEUROSCI.2238-19.2020.video.1

### Mitral cell GCaMP6f imaging confirms broad chemosensory integration

**Figure 2.**
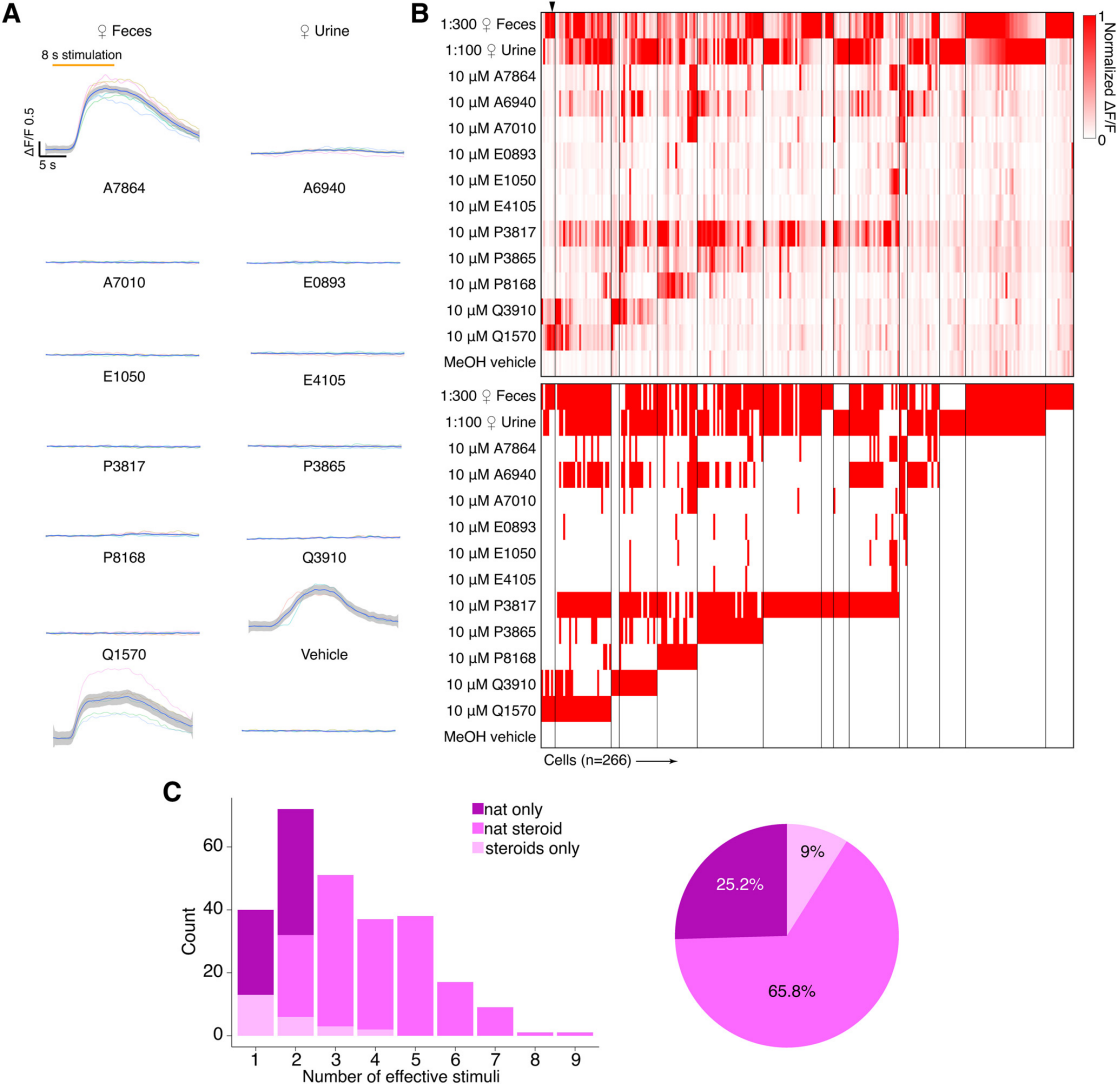
Chemosensory tuning of MCs. ***A***, Averaged response traces from an example MC. Traces were smoothed by local polynomial regression fitting. The shaded regions represent 95% confidence intervals. ***B***, Top, Heat map plot of normalized ΔF/F for 266 MCs. Bottom, Binary heat map plot of MC responsiveness; red tiles indicate a stimulus response that passed statistical criteria. ***C***, Left, Histogram showing the number of effective stimuli per cell. Recorded cells are classified into three color-coded groups based on their responsivity. Right, Pie chart showing the composition of recorded MC populations.

AOB interneurons are principally excited by glutamatergic sensory input from AOB MCs (Brennan and Keverne, 1997; Taniguchi and Kaba, 2001). The tuning of AOB MCs to a similar panel of chemosensory stimuli has been characterized using extracellular single-unit recordings (Meeks et al., 2010). However, since the GCaMP6f imaging platform represents a new approach, we first wanted to investigate the tuning properties of genetically defined MCs and compare these measurements to previous results (Fig. 2). Virally driven GCaMP6f fluorescence in *Pcdh21*-cre mice was observed in cell bodies and apical dendrites of MCs spreading through the ECL and the glomerular layer (Fig. 1*B*). We focused our recordings on GCaMP6f-positive somas, which were all located below the AOB glomerular layer (>70 µm from the AOB surface).

We recorded chemosensory activities of 266 AOB MCs(Fig. 2*B*), a cohort more than twofold larger than previous electrophysiological studies (Hendrickson et al., 2008; Ben-Shaul et al., 2010; Meeks et al., 2010; Tolokh et al., 2013). Consistent with previous results, dilute female mouse urine and feces stimulated strong global activity that began soon after stimulus delivery. Stimulation-evoked ΔF/F typically reached a peak within the first 2 s of an 8 s VNO stimulus delivery and displayed slow decay kinetics (decay time, 8–12 s after the peak). Because the decay kinetics of GCaMP6f (Chen et al., 2013) are much faster than previous measurements of spike frequency decay, the slowness of GCaMP6f offset times likely reflects the slow cessation of spiking activity in AOB MCs (Luo et al., 2003; Wagner et al., 2006; Hendrickson et al., 2008; Ben-Shaul et al., 2010; Meeks et al., 2010; Mohrhardt et al., 2018). Of the 266 MCs we studied, 242 (91.0%) responded to at least one of the naturalistic stimuli, 199 (74.8%) responded to at least one monomolecular sulfated steroid ligand, and 125 (47.0%) were responsive to at least two sulfated steroids (Fig. 2*A*). We also observed a substantial number (24 of 266; 9%) of Pcdh21^+^ cells that were exclusively responsive to one or more sulfated steroids (Fig. 2*C*). Analysis of MC stimulus responses revealed stereotyped patterns of steroid sensitivity that were consistent with previous spiking-based measurements, suggesting that MC GCaMP6f measurements accurately reflect MC activity (Fig. 2*C*; Meeks et al., 2010).

### AOB juxtaglomerular cells show a slight bias toward naturalistic stimuli

The activity of AOB MCs is shaped at multiple levels by inhibitory interneurons. The first stage of MC inhibition occurs in the glomerular layer, where AOB JGCs reside and release GABA onto MC dendrites and VSN presynaptic terminals (Mohrhardt et al., 2018). Because there have not been any systematic recordings of AOB interneuron tuning, we first sought to measure tuning in a general population of AOB GABAergic interneurons. We therefore expressed GCaMP6f in AOB interneurons by stereotaxically injecting AAV9.CAG.Flex.GCaMP6f into the AOB of *Gad2*-IRES-Cre transgenic mice (Taniguchi et al., 2011). A large population of neurons and dendritic arbors in the AOB glomerular layer and the superficial external cellular layer was strongly labeled and visible under the 2-photon microscope (Movie 2). The density of GCaMP6f labeling in the deeper ECL, where EGC somas and IGC dendrites reside, paradoxically precluded the identification of well resolved neuronal recordings. However, neurons in the GL and superficial ECL were readily observed that had small soma size (∼10 µm) and compact dendrites that ramified within the glomerular layer, which is consistent with anatomic descriptions of JGCs (Larriva-Sahd, 2008).

Movie 2. An *ex vivo* 2-photon GCaMP6f Ca^2+^ imaging video 18X speed) showing Gad2^+^ JGCs activated by VNO chemosensory stimulation. 10.1523/JNEUROSCI.2238-19.2020.video.2

**Figure 3.**
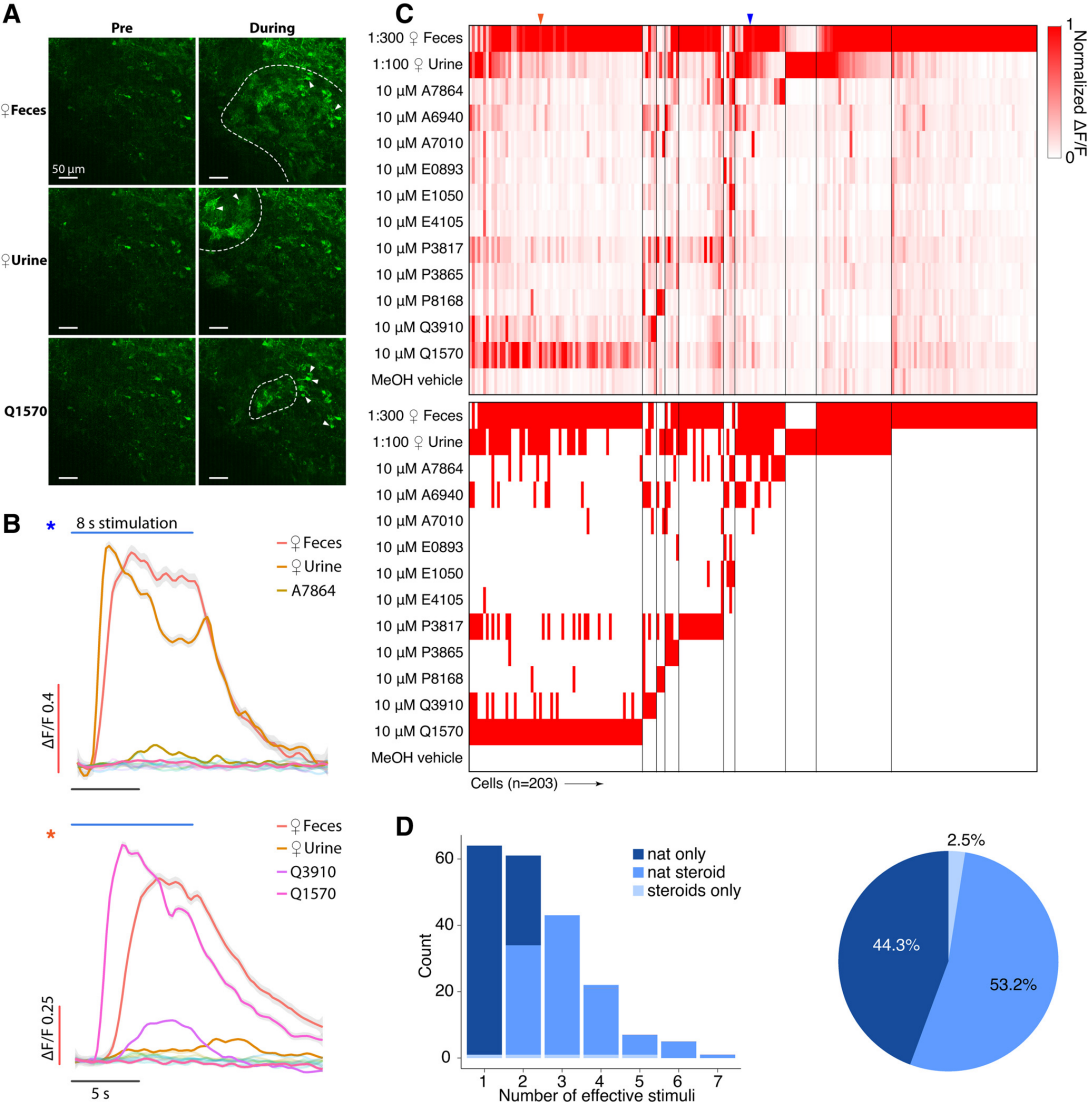
***A***, Example GCaMP6f images of the AOB Gad2^+^ JGCs under chemostimulation. Activated glomeruli are circumscribed by dashed lines. Scale bar, 50 μm. ***B***, Averaged ΔF/F traces for three example JGCs. Traces were smoothed by local polynomial regression fitting. The shaded regions indicate 95% confidence intervals. Colored asterisks indicate the identities of cells in ***C***. ***C***, Top, Heat map of normalized ΔF/F for 203 JGCs. Bottom, Heat map of JGC stimulus responsiveness. Colored arrowheads indicate the recordings presented in ***B***. ***D***, Left, Histogram showing the number of effective stimuli per cell. Recorded cells were classified into three color-coded groups by their selectiveness. Right, Pie chart showing the composition of recorded JGC responsivities.

JGCs reside in the glomerular layer and sense glutamate released by VSN axons and MC dendrites (Jia et al., 1999; Castro et al., 2007). As with MCs, following VNO chemosensory stimulation we observed large, reliable GCaMP6f responses over multiple randomized trials (Movie 2). Dilute BALB/c mouse feces and urine were the two most potent JGC activators. A total of 198 of 203 (97.5%) recorded JGCs showed reliable responses toward female mouse feces or urine (Fig. 3*B*,*C*). In the JGC dataset, 300-fold diluted female mouse feces triggered stronger global activity than 100-fold diluted female mouse urine (Fig. 3*B*), which may at least be a partial consequence of the most accessible imaging region being in the lateral/anterior quadrant of the AOB. Of 113 (55.7%) sulfated steroid-responsive JGCs, 93 (45.8%) were responsive to no more than two different sulfated steroids and 9 (4.4%) were responsive to more than four sulfated steroids (Fig. 3*C*). The response patterns of AOB JGCs to this panel were largely similar to MCs, but a slightly lower percentage of JGCs responded to both naturalistic stimuli and monomolecular ligands than AOB MCs (Figs. 2*C*, 3*C*), suggesting that, as expected based on their restricted glomerular innervation patterns, JGCs perform less excitatory integration than MCs.

### EGC GCaMP6f imaging indicates remarkably sparse chemosensory tuning

**Figure 4.**
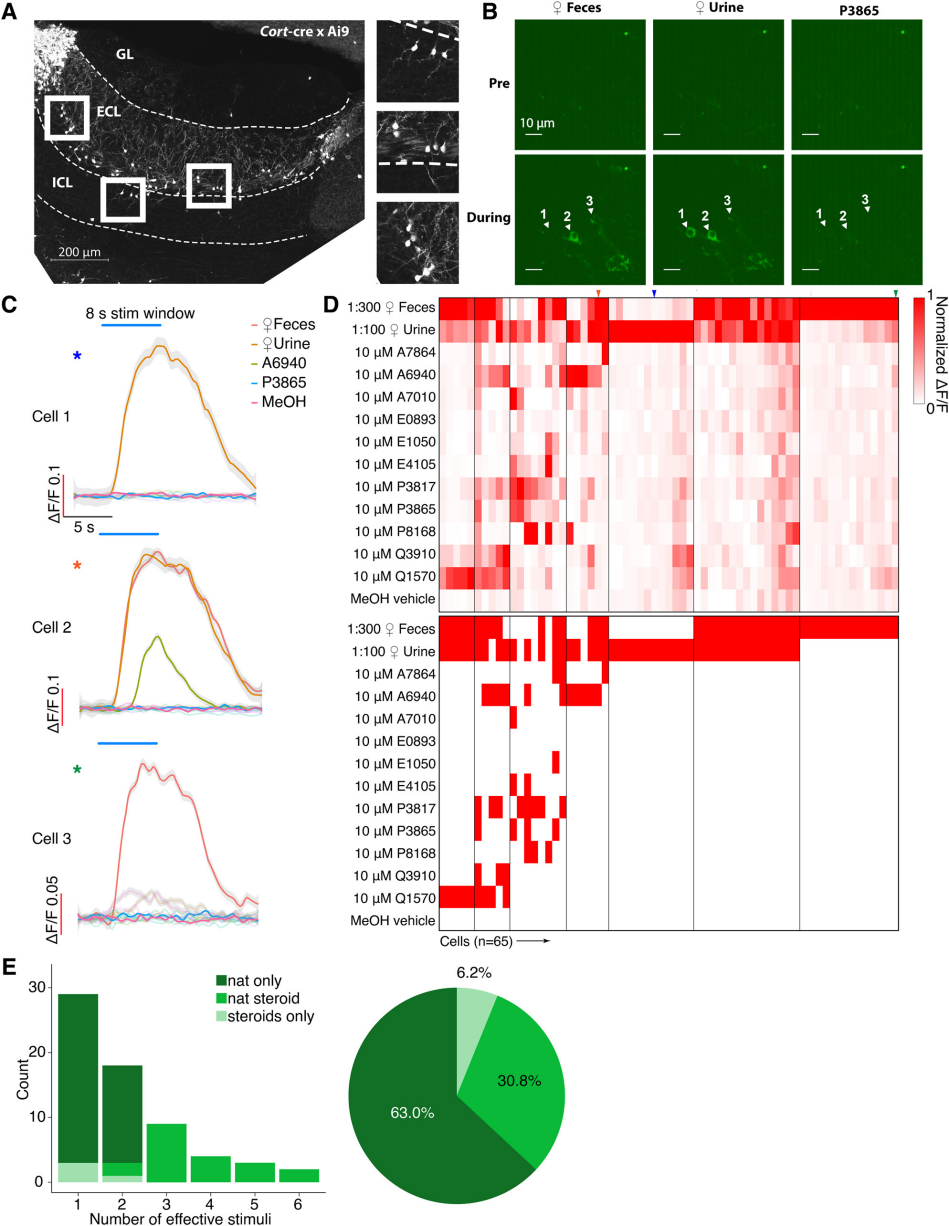
***A***, AOB Sagittal section from a *Cort*-T2A-Cre transgenic mouse mated to a Cre-dependent tdTomato reporter line. Scale bar, 200 μm. ***B***, Raw GCaMP6f images from three example EGCs. Scale bar, 10 μm. ***C***, Averaged ΔF/F traces of the three cells shown in ***B***. Traces were smoothed by local polynomial regression fitting. Shaded regions represent 95% confidence intervals. Colored asterisks indicate the identities of cells in ***D***. ***D***, Top, Heat map of normalized ΔF/F for 65 recorded EGCs. Bottom, Binary heat map of EGC stimulus responsiveness; red tiles represent the pairs that passed the statistical criteria. Colored arrowheads indicate the cells and recordings presented in ***B***, ***C***. ***E***, Left, Histogram of EGC responsivity, with tuning classified into three color-coded subgroups. Right, Pie chart of EGC response classes.

In primary chemosensory circuits, a common inhibitory motif involves broadly integrating interneurons that perform divisive normalization or gain scaling (Wilson et al., 2012; Kato et al., 2013; Jeanne and Wilson, 2015). In the MOB, PV-EPL interneurons have been shown to perform these functions, but it is unknown whether analogous cells exist in the AOB. EGCs seemed well matched to the morphologic and physiological features of MOB PV-EPL neurons (Larriva-Sahd, 2008; Maksimova et al., 2019). Many EGCs are labeled in *Cort*-cre transgenic mice (Maksimova et al., 2019), a fact that we exploited here to specifically target EGCs for viral infection (Fig. 4*A*). We drove GCaMP6f expression in *Cort^+^* EGCs via AAV9.CAG.Flex.GCaMP6f injection into the AOB, followed by 2-photon Ca^2+^ imaging in *ex vivo* preparations.

GCaMP6f expression was concentrated in the AOB ECL (>80 µm from the AOB surface), but basal fluorescence intensity was extremely low compared with AOB cells infected with AAVs in both *Pcdh21*-cre and *Gad2*-cre mice (principal neurons and JGCs, respectively). Baseline GCaMP6f intensity was so low in most infected EGCs that they were not detectable above background until the AOB was activated by VNO stimulation (Materials and Methods; Fig. 4*B*). Identified GCaMP6f-expressing EGCs had small soma size, expansive arborizations, and, in case baseline GCaMP6f fluorescence was high (presumably due to loss of membrane integrity/cell death), we observed large arborizations dense in synaptic spines or gemmules, all of which were consistent with previous descriptions of EGCs (Larriva-Sahd, 2008; Maksimova et al., 2019).

We next investigated the chemosensory tuning properties of Cort^+^ EGCs. To our surprise, these EGCs showed evidence of extremely sparse, rather than broad, tuning to the panel of chemosensory cues (Fig. 4*D*,*E*, Movie 3). Of the 65 recorded EGCs, 41 (63.0%) were activated only by naturalistic stimulation but not by sulfated steroids. Just 24 (37.0%) of 65 Cort^+^ EGCs were responsive to sulfated steroids at all, with only 4 (6.2%) of the recorded cells exclusively activated by sulfated steroids (Fig. 4*D*,*E*). This small population of cells showed broad sulfated steroid tuning, but also showed above-normal baseline fluorescence and spontaneous activity, perhaps suggesting that these cells may have been unhealthy (or are perhaps members of a rare Cort^+^ cell subtype). These experiments indicated that Cort^+^ EGCs have unique features that keep basal GCaMP6f fluorescence low and revealed that EGCs are sparsely tuned to chemical cues. This result was contrary to our hypothesis that EGCs, like PV-EPL interneurons in the MOB, would be more broadly tuned than their upstream MC inputs.

Movie 3. An *ex vivo* 2-photon GCaMP6f Ca^2+^ imaging video 18X speed) showing Cort^+^ EGCs activated by VNO chemosensory stimulation. 10.1523/JNEUROSCI.2238-19.2020.video.3

### Chemosensory tuning comparisons among MCs, JGCs, and EGCs

**Figure 5.**
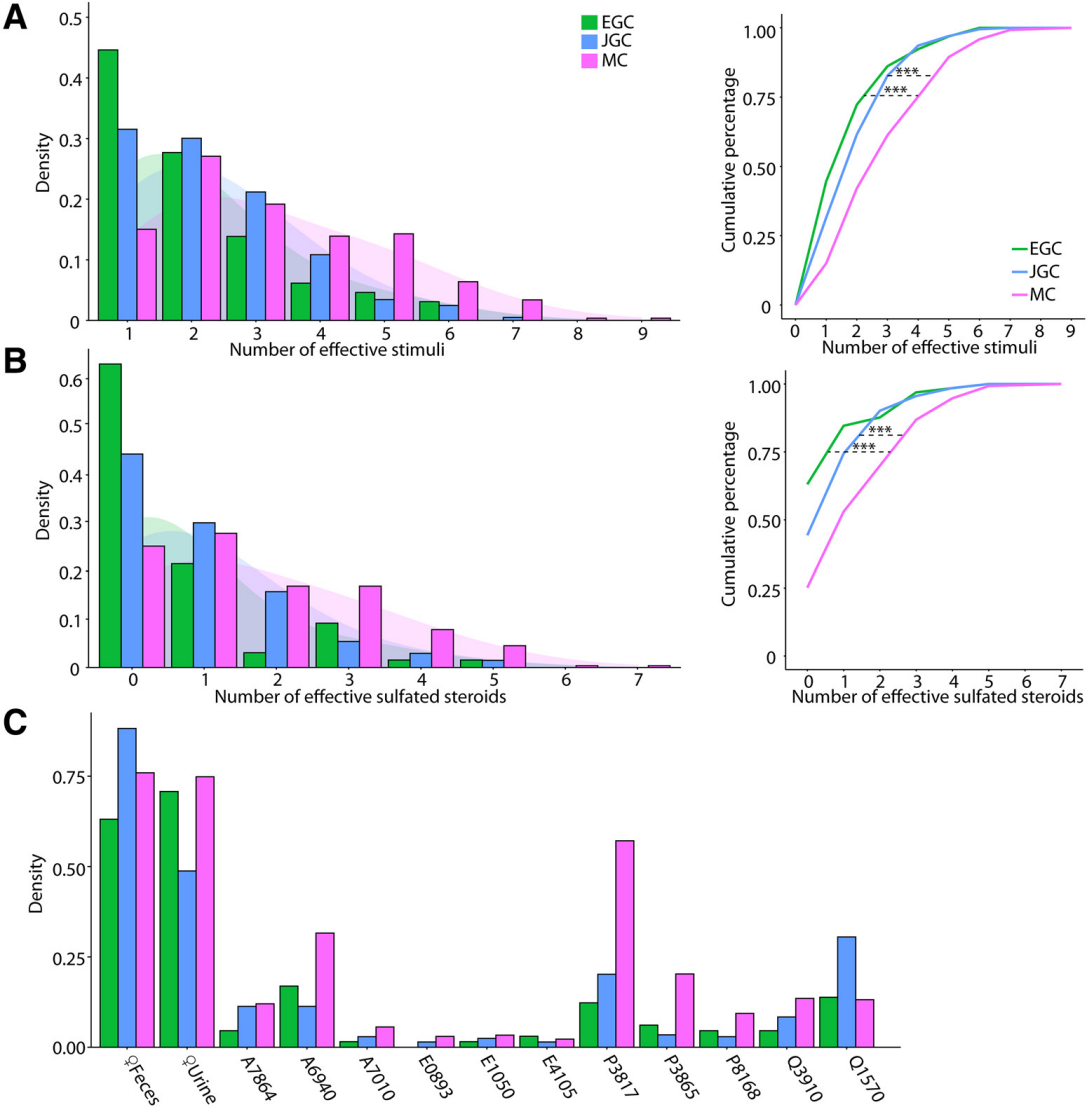
***A***, Left, Histograms of the number of effective stimuli for EGCs, JGCs, and MCs. The shaded regions indicate Gaussian kernel densities. Right, Triple asterisks indicate statistically significant differences between groups (EGC vs MC, *p* = 1.5e-4; EGC vs JGC, *p* = 0.37; MC vs JGC, *p* = 4.9e-5, K-S test) ***B***, Same as in ***A***, with dilute urine and feces stimuli excluded. Triple asterisks indicate statistically significant differences between groups (EGC vs MC, *p* = 6.1e-7; JGC vs MC, *p* =3.3e-5; EGC vs JGC, *p* = 0.063, K-S test). ***C***, Percentage of EGCs, JGCs, and MCs that responded to each stimulus in the panel.

To more directly investigate cell type-specific tuning in the AOB, we assessed tuning breadth to all stimuli and monomolecular steroids across all of the cell types studied (see “Materials and Methods”; Fig. 5). The distributions of effective stimuli with (Fig. 5*A*) or without (Fig. 5*B*) naturalistic stimuli per cell indicated that MCs demonstrated the broadest tuning to this panel of chemosensory cues, with 103 of 266 (38.7%) being responsive to no less than four stimuli (Fig. 5*A*), and 125 of 266 (47.0%) are responsive to no less than three sulfated steroids. In contrast, the majority of EGCs (47 of 65; 72.3%) were responsive to two or fewer stimuli and 41 of 65 (43.1%) were not responsive to any sulfated steroids tested. Gad2^+^ JGCs demonstrated intermediate tuning in cases both with and without naturalistic stimuli. The broadness of MC tuning is consistent with heterotypic integration of VNO inputs by AOB MCs (Wagner et al., 2006; Meeks et al., 2010). When natural ligand blends were included, the cumulative distributions of effective stimuli showed significant tuning differences between each interneuron type and MCs (EGC vs JGC, *p* = 0.37; EGC vs MC, *p* = 1.5e-4; JGC vs MC, *p* = 4.9e-5; Kolmogorov–Smirnov test; Fig. 5*B*, left). When naturalistic stimuli were excluded (Fig. 5*B*, right), these effects were even more pronounced (EGC vs JGC, *p* = 0.063; EGC vs MC, *p* = 6.1e-7; JGC vs MC, *p* = 3.3e-5; Kolmogorov–Smirnov test). EGCs, JGCs, and MCs have similar distribution patterns across monomolecular steroid stimuli (Fig. 5*C*). For example, A6940, P3817, Q1570, and Q3910 activated the largest number of neurons in all three cell populations, while A7010, E0893, E1050, and E4105 activated the least number of neurons. Overall, the tuning patterns observed for monomolecular sulfated steroids in all cell types were consistent with previous studies (Meeks et al., 2010; Turaga and Holy, 2012; Hammen et al., 2014).

JGC and EGC responses to natural ligand blends were overrepresented compared with MCs (Figs. 2*C*, 3*C*, 4*E*). Specifically, 63% of EGCs (Fig. 4*E*), and 44.3% of JGCs (Fig. 3*C*) responded exclusively to natural ligand blends, compared with 25.2% for MCs (Fig. 2*C*). Conversely, 9% of MCs are exclusively activated by sulfated steroids, compared with 6.2% of EGCs and 2.5% of JGCs. These differences in the proportion of responsive neurons may reflect complex network effects. However, they may more simply reflect differences in activation thresholds; previous studies indicated that MCs have higher signal-to-noise ratios and lower effective thresholds for activation than their VSN inputs (Meeks et al., 2010).

### EGCs and MCs show similar GCaMP6f-to-spiking relationships during simulated sensory activity

The observation that he chemosensory tuning of EGCs is much sparser than MCs was contrary to our initial hypothesis. One possible explanation for this observation is that EGC GCaMP6f signals more weakly reflect spiking activity in EGCs compared with MCs. To study GCaMP6f-to-spiking relationships, we first performed 2-photon guided whole-cell patch-clamp recordings on EGCs and MCs while recording their GCaMP6f signals in acute brain slices. We first found that resting membrane potentials in EGCs (–86.0 ± 1.0 mV; *N* = 22) were significantly hyperpolarized compared with those of MCs (−63.7 ± 1.0 mV; *n* = 18), confirming earlier results (Gorin et al., 2016; Maksimova et al., 2019).

**Figure 6.**
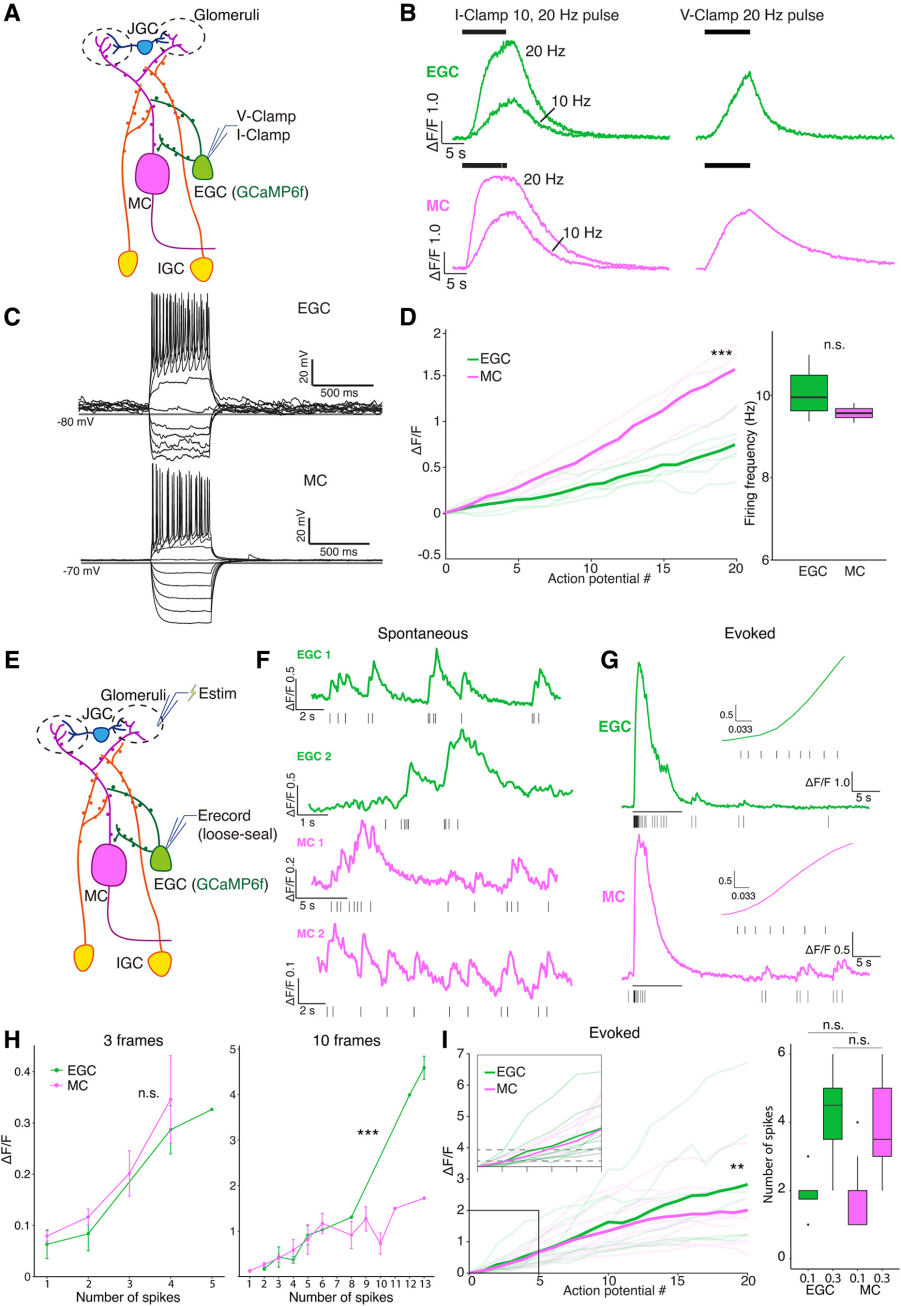
***A***, Diagram of the whole-cell patch-clamp setup. ***B***, Example GCaMP6f fluorescence response to artificial current injection in current clamp (I-Clamp, left) and voltage clamp (V-Clamp, right). ***C***, Example responses of EGCs and MCs in response to current injection steps. ***D***, Left, The ΔF/F-number-of-spikes relationship under ∼10 Hz firing. Triple asterisks indicate statistical significance (*p* = 5.06e-36, main effect of cell type; two-way ANOVA). Right, Firing rate of EGC and MC. n.s.: not statistically significant (Student's *t* test, *p* = 0.35). ***E***, Diagram of loose-seal cell-attached recording and glomerular layer stimulation. Estim, Theta glass stimulating electrode; Erecord, loose-seal recording electrode. ***F***, Example loose-seal cell-attached recording traces from two EGCs and two MCs show clear fluorescence increases following single spontaneous spikes. ***G***, Example evoked fluorescence traces of an EGC and an MC. The black bar indicates the stimulation window. The insets are enlarged views of the first ∼0.25 s after stimulation onset. ***H***, ΔF/F at the 3 and 10 frames immediately after the onset of spike 1, plotted against the number of spikes that occurred in this time window. Triple asterisks indicate statistical significance; n.s. indicates lack of statistical significance(3 frames, *p* = 0.65; 10 frames, *p* = 2.04e-6, main effect of cell type, two-way ANOVA). ***I***, Left, The ΔF/F-to-spiking relationship measured by loose-seal cell-attached recording. Double asterisk indicates statistical significance (*p* = 4.1e-3, main effect of cell type, two-way ANOVA). Right, The number of action potentials required to pass ΔF/F = 0.1 (Student's *t* test, *p* = 0.91) and ΔF/F = 0.3 (Student's *t test*, *p* = 0.66). n.s indicates lack of statistical significance.

We investigated the GCaMP6f responses to pulse stimulation in both current-clamp and voltage-clamp modes (Fig. 6*A–D*). In current clamp, EGCs and MCs reached comparable peak ΔF/F values during 10 s, 20 Hz somatic pulse trains (Fig. 6*B*). In both voltage and current clamp, MCs showed faster rising dynamics than EGCs (Fig. 6*B*). In voltage clamp, most MCs (20 of 22) showed a sigmoidal ΔF/F stimulus–response relationship (Movie 4), whereas many EGCs (6 of 17) failed to reach a plateau at all (Fig. 6*B*, Movie 5).

Movie 4. Pcdh21+ MC GCaMP6f Ca^2+^ imaging coupled with 10 s, 20 Hz depolarization pulse train in voltage clamp. 10.1523/JNEUROSCI.2238-19.2020.video.4

Movie 5. Cort^+^ EGC GCaMP6f Ca^2+^ imaging coupled with 10 s, 20 Hz depolarization pulse train in voltage clamp. 10.1523/JNEUROSCI.2238-19.2020.video.5

We further tested the GCaMP6f performance with step current injections in current-clamp mode. Standardized current injection ramps elicited comparable maximal spike frequencies in EGCs (27 ± 1.53 Hz, *N* = 6) and MCs (25 ± 3.70 Hz, *N* = 4; Fig. 6*C*). We measured changes in GCaMP6f intensity during step current injections that evoked ∼10 Hz firing for 10 s (MC: 9.64 ± 0.131 Hz, *N* = 4; EGC: 10.07 ± 0.256 Hz, *N* = 6; *p* = 0.11, Student's *t* test; Fig. 6*D*). In these conditions, both cell types reached equally high peak ΔF/F (MC, 3.68 ± 0.267; EGC, 3.27 ± 0.692; Student's *t* test, *p* = 0.63). Analysis of the GCaMP6f-to-spiking relationship for the first 20 spikes in these trials showed that both MCs and EGCs showed a nearly linear ΔF/F relationship (Fig. 6*D*). However, MCs demonstrated a faster increase in ΔF/F, as evidenced by a significantly larger GCaMP6f-to-spiking slope in MCs compared with EGCs (*p* = 5.06e^−36^, main effect of cell type; *p* = 9.79e^−12^, cell type– spike number interaction, two-way ANOVA). Thus, whole-cell patch clamp-based approaches indicated more robust GCaMP6f-to-spiking performance in MCs than in EGCs.

Patch-clamp-based approaches for evaluating GCaMP6f-to-spiking relationships are common (Dana et al., 2019; Inoue et al., 2019; Wei et al., 2019), but have several important caveats. For example, whole-cell patch clamp disrupts cytoplasmic Ca^2+^ buffering environment. Moreover, stimulating spiking through the somatic patch-clamp electrode does not activate these cells via the natural progression of synaptic excitation experienced *in vivo* (or the *ex vivo* preparation). To evaluate GCaMP6f performance during more natural waves of synaptic activation, we performed simultaneous GCaMP6f Ca^2+^ imaging and loose-seal cell-attached recordings while electrically stimulating the AOB glomerular layer (EGC, *N* = 8; MC, *N* = 12; Movies 6, 7, Fig. 6*E*). In these recordings, both MCs and EGCs sometimes fired spontaneously, eliciting clear GCaMP6f responses (Fig. 6*F*). When glomerular layer stimulation was applied, we recorded stimulus-evoked action potential trains in MCs and EGCs of equivalent frequencies (MC: 8.76 ± 1.87 Hz, N = 12; EGC: 12.63 ± 3.50 Hz; *N* = 8). Unlike in whole-cell patch-clamp experiments, these recordings showed rapid GCaMP6f increases in both cell types (Fig. 6*G*). We analyzed the GCaMP6f response following the initial stimulus-evoked spike by plotting the GCaMP6f amplitude achieved versus the number of spikes within a fixed window after spike 1. We evaluated GCaMP6f responses at 3 frames (∼100 to 132 ms) and 10 frames (∼333 to 365 ms) to show the short-term and longer-term development of the evoked signal (Fig. 6*H*). Within three frames, EGCs and MCs showed no difference (EGC, 19 trials of 8 cells; MC, 37 trials of 12 cells; *p* = 0.65, main effect of cell type; *p* = 0.90, cell type–spike number interaction, two-way ANOVA). Within 10 frames, EGCs started to develop a stronger cumulative effect than MCs (EGC, 18 trials of 7 cells; MC, 36 trials of 12 cells; *p* = 2.04e^−6^, main effect of cell type; *p* = 2.1e^−12^, cell type–spike number interaction, two-way ANOVA). The relationship between the first 20 spikes and ΔF/F produced similar results (*p* = 4.1e^−3^, main effect of cell type; *p* = 0.99, cell type–spike number interaction, two-way ANOVA). Both MCs and EGCs reached the 30% ΔF/F criterion used to determine responsiveness in the *ex vivo* imaging experiments (Figs. 2–5) after approximately four spikes (Fig. 6*H*; EGC: 4.13 ± 0.52, *N* = 8; MC: 3.83 ± 0.39, *N* = 12; *p* = 0.66, Student's *t* test), and the 10% ΔF/F criterion only after approximately two spikes (EGC: 1.88 ± 0.23, *N* = 8; MC: 1.92 ± 0.26, *N* = 12; *p* = 0.91, Student's *t* test). In summary, these experiments show that EGCs and MCs, despite clear differences in GCaMP6f-to-spiking characteristics in the whole-cell patch-clamp configuration, demonstrate similar GCaMP6f responses to spiking when the AOB circuit is engaged. As such, these data suggest that differences in GCaMP6f performance between MCs and EGCs cannot explain the tuning sparseness we observed in EGCs in *ex vivo* preparations (Figs. 2–5).

Movie 6. Cort^+^ EGC loose-seal cell-attached recording with simultaneous GCaMP6f Ca^2+^ imaging. 10.1523/JNEUROSCI.2238-19.2020.video.6

Movie 7. Pcdh21+ MC loose-seal cell-attached recording with simultaneous GCaMP6f Ca^2+^ imaging. 10.1523/JNEUROSCI.2238-19.2020.video.7

### *Ex vivo* EGC whole-cell recordings reveal broad subthreshold responsiveness

**Figure 7.**
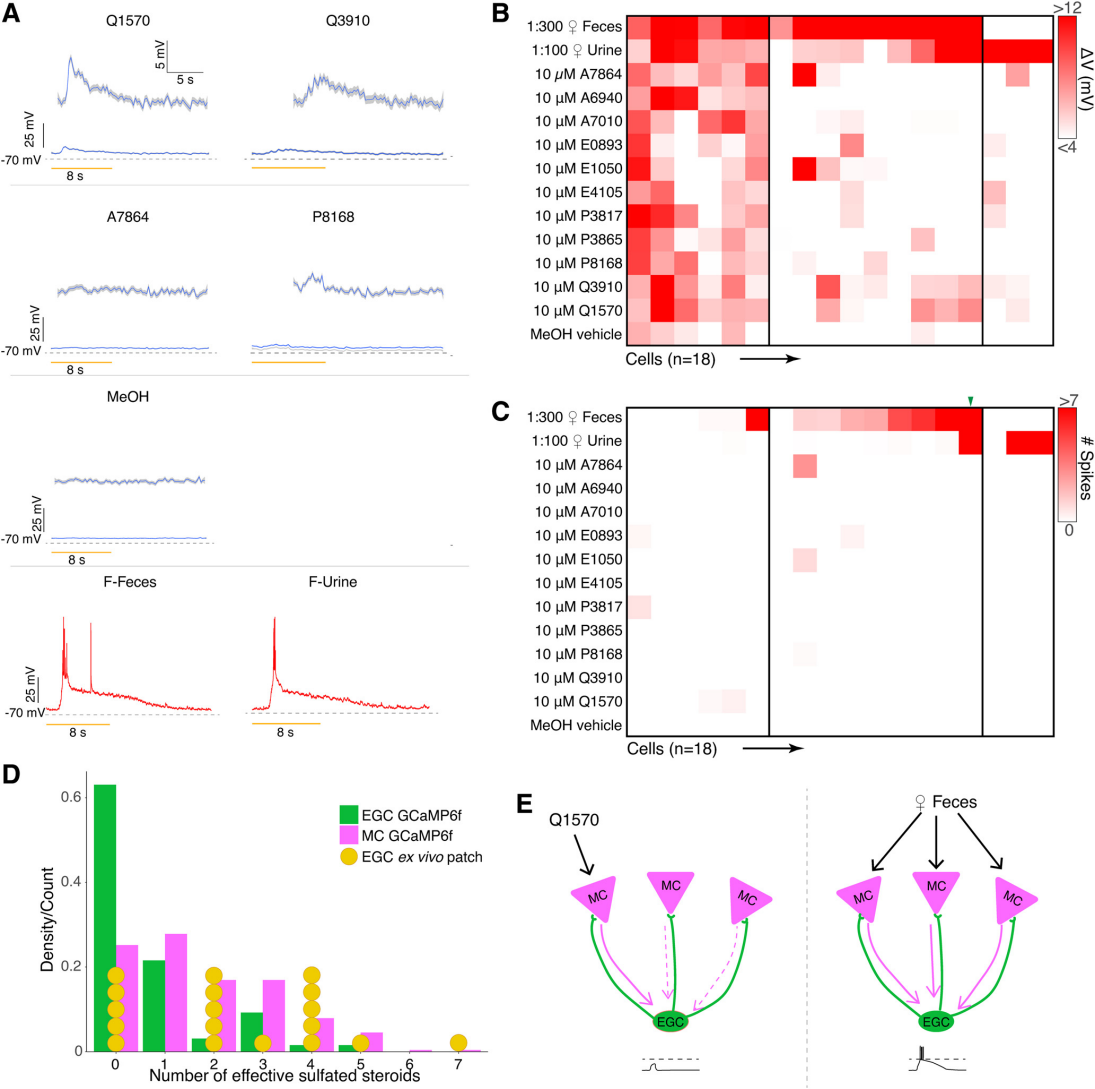
***A***, Average voltage traces of an example EGC in the *ex vivo* preparation during VNO stimulation. Subthreshold responses activities are enlarged in the insets. Q1570, Q3910, and P8168 reliably elicit subthreshold EPSPs. Urine and fecal extracts cause action potentials. ***B***, Heatmap of average voltage changes of 18 recorded EGCs. ***C***, Heatmap of the spiking responses of the same 18 EGCs. ***D***, Comparison between responsiveness measured via Ca^2+^ imaging and patch clamp. ***E***, Threshold integration model of MC–EGC connectivity.

Measuring the relationships between spiking and ΔF/F in MCs and EGCs did not account for the sparseness of observed EGC tuning. Moreover, ΔF/F-to-spiking estimates were made using artificial spike-inducing protocols that may not reflect spiking conditions in the *ex vivo* chemosensory tuning experiments. Another possible explanation for EGC tuning sparseness could be the highly hyperpolarized resting membrane potentials of EGCs, which our data and previous studies indicated are ∼15 to 20 mV hyperpolarized compared with other AOB neurons (Maksimova et al., 2019). This extreme resting hyperpolarization could prevent EGC spiking in all but the strongest stimulation conditions, potentially preventing the observation of robust GCaMP6f signals. We therefore performed 2-photon fluorescence-guided whole-cell patch-clamp recordings on Cort^+^ EGCs in the *ex vivo* preparation (Fig. 7). We performed these experiments in *Cort-*T2A-Cre mice crossed to a cre-dependent tdTomato reporter line (Madisen et al., 2010), which improved our ability to identify EGC somata at rest. Using techniques similar to those in the study by Häusser and Margrie (2014), we achieved the whole-cell configuration, then maintained each cell in current clamp near its initial resting potential via DC current injection. EGC resting membrane potentials in the *ex vivo* preparations, measured immediately after break-in, were depolarized compared with AOB slices (−63.7 ± 2.0 mV, *n* = 18). The reasons for the discrepancy were not clear, given that the internal and external solutions were identical to those in slice experiments. Nevertheless, we decided to maintain patched EGCs at these relatively depolarized potentials because they more likely reflected the state of EGCs in this *ex vivo* preparation (i.e., during GCaMP6f imaging experiments; Figs. 2, 4). Importantly, the observation that EGCs had relatively depolarized resting membrane potentials in the *ex vivo* preparation suggests that, if anything, EGCs in this preparation might be much closer to action potential threshold than was suggested by resting potentials measured in slice experiments.

We recorded EGC responses while we stimulated the VNO with the same panel of chemical cues used in our GCaMP6f recordings (Fig. 7). Consistent with our GCaMP6f results, action potentials were reliably triggered in EGCs following VNO stimulation with natural ligand blends (dilute mouse urine or feces), but rarely by any monomolecular ligands in the stimulation panel (Fig. 7*A*). The spiking responses to peripheral stimulation typically included burst firing early in the stimulus presentation (11.90 ± 2.56 Hz, *n* = 20) followed by a long-lasting subthreshold decay period that extended well beyond the stimulation window (mean 90–10% decay time, 9.70 ± 0.44 s; *n* = 76 cell–stimulation pairs). The depolarization decay kinetics are comparable for naturalistic stimuli (mean 90–10% decay time, 10.26 ± 0.49 s; *n* = 31 cell–stimulation pairs) and monomolecular ligands (mean 90–10% decay time, 9.30 ± 0.65 s; *n* = 45 cell–stimulation pairs). Importantly, we readily observed broad subthreshold responses to many monomolecular steroid ligands (Fig. 7*A*). These subthreshold responses were tightly coupled to the onset of stimulation and were reliable across repeated trials (Fig. 7*A*). The overall envelope of depolarization was consistent with the time course of activation of MC GCaMP6f activity (Figs. 1, 2; mean, 90–10% decay time, 8.09 ± 0.06 s; *n* = 978 cell–stimulation pairs) and previous studies (Wagner et al., 2006; Hendrickson et al., 2008; Meeks et al., 2010; Yoles-Frenkel et al., 2018).

Investigating the patterns of EGC subthreshold responsiveness revealed much broader MC integration than was indicated by GCaMP6f imaging experiments. Of 18 responsive EGCs, 13 showed only subthreshold activity, without spiking, to VNO stimulation with monomolecular sulfated steroids (Fig. 7*B*; *p* < 0.05 compared with the vehicle control, Wilcoxon rank sum test). Comparing the summed subthreshold and suprathreshold tuning to suprathreshold-only tuning in this group of patched cells revealed the major source of discrepancy between MC and EGCs (Fig. 7*B*,*C*). For example, 4 of the 18 recorded cells, despite clear subthreshold responses to these stimuli, did not spike at all. Of the 14 cells that spiked in response to these stimuli, the majority (9 of 14) spiked only in response to naturalistic ligand blends, in agreement with GCaMP6f-based results (Fig. 4*D*). When we included subthreshold activation in our criteria, the distribution of EGC responsivity was significantly right skewed (broader) than the distribution EGCs determined by the GCaMP6f imaging signal (*p* = 2.2e-4, Kolmogorov–Smirnov test), and became statistically indistinguishable from MCs (*p* = 0.21, Kolmogorov–Smirnov test; Fig. 7*D*). These results help to explain our *ex vivo* Ca^2+^ imaging observations and support the hypothesis that EGCs broadly integrate from MCs (Fig. 7*E*). However, these data also show that Cort^+^ EGCs are only effectively driven to spike by stimuli that elicit spikes in a large number of AOB mitral cells (in this stimulus panel, dilute mouse urine and feces). Collectively, these results suggest that AOB EGCs perform fundamentally different roles in the AOB than PV-EPL interneurons play in the MOB. Though these cells are broad integrators, they are sparsely tuned at the level of spiking, suggesting that their activity is only stimulated in conditions in which a large ensemble of MCs is simultaneously activated.

## Discussion

### Cell type-specific functional studies in the AOB *ex vivo* preparation

Our knowledge of interneuron function in the AOB is generally lacking due to persistent technical challenges to recording from these neurons during VNO stimulation. The *ex vivo* preparation of the early mouse AOS, which allows optical access to the AOB while preserving VNO–AOB functional connectivity, overcomes some of the major hurdles to performing cell type-specific investigations of chemosensory tuning. However, the results of *ex vivo* studies do come with some limitations. For example, the *ex vivo* preparation eliminates the influence of potential feedback neuromodulation from downstream brain areas, which is clearly important for AOB circuit function (Oboti et al., 2018). Despite this significant limitation, by removing some *in vivo* complexities, the *ex vivo* approach has clear advantages for dissecting the basic structure and function of the AOB circuit.

By combining *ex vivo* methods with 2-photon microscopy and genetic tools for cell type-specific manipulation in the nervous system, we were able to perform targeted studies of chemosensory tuning in genetically defined AOB interneuron subsets. The specific cell types explored in this study, namely MCs, JGCs, and EGCs, represent three of the four major neuronal classes (with the remaining major class being the IGCs). Our approach allowed us to produce quantitative comparisons of the stimulus–response characteristics of each cell type, and to do so across multiple randomized stimulus trials to reduce the possible impact of spontaneous activity (Holy et al., 2000; Nodari et al., 2008; Meeks et al., 2010). The utility of this combination of techniques for studying AOB circuit function is thus clear, and the results of these experiments allowed us to reveal key differences in the function of AOB EGCs compared with superficially similar PV-EPL interneurons in the MOB (Kato et al., 2013; Miyamichi et al., 2013).

### AOB EGCs are broadly innervated, but sparsely tuned to chemosensory stimuli

AOB MCs are capable of integrating excitatory input from VSNs that express different sensory vomeronasal receptors (Wagner et al., 2006) and are broadly tuned to sensory input (Meeks et al., 2010). As such, observing a high amount of tuning diversity in MCs to both naturalistic stimulation and a well characterized panel of monomolecular sulfated steroids (Fig. 2) was expected. The broad and variable patterns of MC tuning adds to a growing list of studies indicating that these cells support the encoding of the identity of a chemosignal-emitting animal (Luo et al., 2003; Hendrickson et al., 2008; Ben-Shaul et al., 2010; Tolokh et al., 2013). The high degree of activation of MCs by the monomolecular sulfated steroid ligands in this panel also further supports the notion that these cells possess higher coding robustness than their VSN inputs, a feature shared by principal neurons in other sensory circuits and species (Bhandawat et al., 2007; Meeks et al., 2010; Zhu et al., 2013).

Our a priori hypothesis was that AOB EGCs were functionally analogous to MOB PV-EPL interneurons, so we were surprised when we observed significantly sparser chemosensory tuning in EGCs compared with their upstream MCs inputs. While many EGCs were reliably activated by female BALB/c mouse urine or fecal extracts, few showed responsiveness to monomolecular ligands (Fig. 4). This result was counterintuitive, given that EGCs have extensive spinous dendritic arborizations in the ECL and receive a constant barrage of strong glutamatergic excitation from MCs, even in the absence of VSN activation (Maksimova et al., 2019). We investigated whether differential GCaMP6f signaling in EGCs and MCs could contribute to this observation. To do so, we used whole-cell patch-clamp and loose-seal cell-attached recording (Fig. 6). Though discrepancies in GCaMP6f reporting efficiency were observed in the whole-cell patch-clamp configuration, loose-seal recordings during glomerular layer stimulation, which more closely mimics natural AOB stimulation, indicated no differences between these cell types (Fig. 6*E–I*). Our results indicate that our criteria for responsiveness are achieved with 2 or more spikes in MCs and EGCs (Fig. 6*G*–*I*). This approach should therefore achieve a low false-negative rate, but it is worth noting that single-spike responses (in both MCs and EGCs) may not have been detected. These results provide important evidence that differences in GCaMP6f signals across AOB MCs and EGCs cannot account for the observed tuning sparseness in EGCs.

EGCs were previously noted for having extremely hyperpolarized resting membrane potentials (Maksimova et al., 2019), which suggested that EGCs may possess very high thresholds for action potential generation from resting states. Whole-cell patch-clamp studies of EGCs in the *ex vivo* preparation revealed that, despite using the same internal and external solutions as in slice experiments, EGC resting membrane potentials were more depolarized in the *ex vivo* preparation than in slices. This could be the result of incomplete perfusion of the relatively low [K^+^]_o_ in the aCSF (2.5 mm), or perhaps due to an overall higher excitatory tone in this preparation (or both). Importantly, these resting membrane potential measurements were made in the same conditions in which EGC GCaMP6f chemosensory tuning measurements were made, suggesting that EGC resting hyperpolarization has a less dramatic impact on tuning sparseness than expected based on slice results. In *ex vivo* whole-cell recordings, we observed rich subthreshold sulfated steroid-evoked activity, but little spiking except in response to stimulation with urine or feces (Fig. 7). Thus, despite mildly depolarized resting membrane potentials in the *ex vivo* preparation, EGCs demonstrate resistance to action potential generation unless a very large MC ensemble is simultaneously active (as is the case when the VNO is stimulated with mouse urine and feces). Physiologic mechanisms that could contribute to high EGC thresholds for spiking could include selective expression of leak channels on EGC dendrites and shunting inhibition by other interneurons (Chamberland and Topolnik, 2012). Future studies will be needed to investigate the source of high EGC spiking thresholds.

It is worth also noting that although the *Cort-*T2A-cre transgenic mice used in these studies selectively label AOB EGCs, the Cort^+^ EGC population likely represents a fraction of the total EGC population (Maksimova et al., 2019). As such, it is possible that the chemosensory tuning we observed represents a specific subgroup of Cort^+^ EGCs. This seems unlikely, given that no differences were found among EGC morphologies, intrinsic features, and synaptic features across several transgenic lines that label these cells (Maksimova et al., 2019). Also, although Cort^+^ EGC labeling spans the anterior and posterior AOBs, our optical recordings were largely confined to portions of the anterior AOB where responsiveness to the cues in our panel is most prominent (Meeks et al., 2010; Doyle et al., 2014). As such, it may be the case that EGCs in the posterior AOB have different tuning qualities than are indicated in this study.

### Implications for models of AOB information processing

The complex physiological properties of EGCs and their chemosensory tuning are becoming clearer, but the impacts of EGC activation on MC function remain unclear. Many EGCs are labeled in *Gad2*-IRES-cre transgenic lines (Maksimova et al., 2019), consistent with a GABAergic phenotype. EGCs are axonless and have spinous dendrites that closely appose MC dendrites, and AOB MCs are known to form reciprocal dendrodendritic synapses with other AOB interneurons (Jia et al., 1999; Taniguchi and Kaba, 2001; Castro et al., 2007; Larriva-Sahd, 2008). Seemingly analogous PV-EPL interneurons in the MOB have been shown to be broadly inhibitory (Kato et al., 2013; Miyamichi et al., 2013; Liu et al., 2019). All of these pieces of evidence point to a reciprocalinhibitory function for EGCs, and future studies will be able to further elucidate the impact of EGCs on MC function and information flow through the AOB.

Regardless of the mechanisms underlying the specific chemosensory tuning features of EGCs, the observation that EGCs rarely spike in the absence of a naturalistic ligand blend has important implications for AOB circuit function. First, these results suggest that EGCs have unique roles in AOB processing that are different from PV-EPL interneurons in MOB. This is not the first observation of seemingly analogous neural types in the MOB and AOB having different physiological properties (Shipley and Adamek, 1984; Jia et al., 1999; Araneda and Firestein, 2006; Wagner et al., 2006; Castro et al., 2007; Smith et al., 2015). In the MOB, PV-EPL interneurons are activated by many monomolecular odorants with low thresholds, resulting in chemosensory tuning that is close to a simple linear addition of the tuning maps of input MCs (Kato et al., 2013). This quality benefits unbiased monitoring of MC activity and supports divisive normalization of MCs based on the overall population response (Kato et al., 2013; Miyamichi et al., 2013; Liu et al., 2019). In contrast, AOB EGCs appear to have extremely high effective thresholds despite receiving synaptic input from many MCs, which may strongly bias their activity away from monomolecular ligands and toward the blends of ligands found in natural excretions (Nodari et al., 2008). Since natural vomeronasal social cues are only known to exist in the form of such complex blends, the difference in monomolecular tuning sparseness between AOB EGCs and MOB PV-EPL interneurons might reflect macroscopic differences in the natural statistics of ligand sampling between these two chemosensory pathways. It may also be the case that MC inhibition by EGCs takes place locally at reciprocal dendrodendritic synapses in a spiking-independent manner, as has been observed in the MOB (Isaacson and Strowbridge, 1998; Schoppa et al., 1998; Chen et al., 2000; Halabisky et al., 2000; Isaacson, 2001; Egger et al., 2005; Huang et al., 2013; Bywalez et al., 2015; Lage-Rupprecht et al., 2018).

AOB EGCs thus appear to be, at a minimum, operating in a manner that is clearly different from PV-EPL interneurons in the MOB, raising questions about their *in vivo* roles in sensory processing. Several studies have reported individual vomeronasal ligands capable of evoking significant behavioral effects through the AOS (Chamero et al., 2007; Haga et al., 2010; Papes et al., 2010). If our results hold for other AOS ligands, it seems unlikely that these particular chemosensory exposures engage EGCs, which may have important implications for information flow through the AOB toward its downstream targets in the limbic system (Martinez-Marcos, 2009; Gutiérrez‐Castellanos et al., 2014; Stowers and Liberles, 2016). In sum, these experiments contribute a wealth of information about chemosensory tuning of specific AOB cell types, adding important quantitative constraints on the role of inhibitory interneurons on AOB circuit function.

## References

[B1] Araneda RC, Firestein S (2006) Adrenergic enhancement of inhibitory transmission in the accessory olfactory bulb. J Neurosci 26:3292–3298. 10.1523/JNEUROSCI.4768-05.2006 16554479 PMC6674102

[B2] Ben-Shaul Y, Katz LC, Mooney R, Dulac C (2010) In vivo vomeronasal stimulation reveals sensory encoding of conspecific and allospecific cues by the mouse accessory olfactory bulb. Proc Natl Acad Sci U S A 107:5172–5177. 10.1073/pnas.0915147107 20194746 PMC2841925

[B3] Bhandawat V, Olsen SR, Gouwens NW, Schlief ML, Wilson RI (2007) Sensory processing in the Drosophila antennal lobe increases reliability and separability of ensemble odor representations. Nat Neurosci 10:1474–1482. 10.1038/nn1976 17922008 PMC2838615

[B4] Braganza O, Beck H (2018) The circuit motif as a conceptual tool for multilevel neuroscience. Trends Neurosci 41:128–136. 10.1016/j.tins.2018.01.002 29397990

[B5] Brennan PA (2001) The vomeronasal system. Cell Mol Life Sci 58:546–555. 10.1007/PL00000880 11361090 PMC11146473

[B6] Brennan PA, Keverne EB (1997) Neural mechanisms of mammalian olfactory learning. Prog Neurobiol 51:457–481. 10.1016/s0301-0082(96)00069-x 9106902

[B7] Brennan PA, Binns EK (2005) Vomeronasal mechanisms of mate recognition in mice. Chem Senses 30 [Suppl 1]:i148–i149. 10.1093/chemse/bjh157 15738084

[B8] Brennan PA, Kendrick KM, Keverne EB (1995) Neurotransmitter release in the accessory olfactory bulb during and after the formation of an olfactory memory in mice. Neuroscience 69:1075–1086. 10.1016/0306-4522(95)00309-7 8848096

[B9] Bywalez WG, Patirniche D, Rupprecht V, Stemmler M, Herz A, Pálfi D, Rózsa B, Egger V (2015) Local postsynaptic voltage-gated sodium channel activation in dendritic spines of olfactory bulb granule cells. Neuron 85:590–601. 10.1016/j.neuron.2014.12.051 25619656

[B10] Cansler HL, Maksimova MA, Meeks JP (2017) Experience-dependent plasticity in accessory olfactory bulb interneurons following male–male social interaction. J Neurosci 37:7240–7252. 10.1523/JNEUROSCI.1031-17.2017 28659282 PMC5546401

[B11] Castro JB, Hovis KR, Urban NN (2007) Recurrent dendrodendritic inhibition of accessory olfactory bulb mitral cells requires activation of group I metabotropic glutamate receptors. J Neurosci 27:5664–5671. 10.1523/JNEUROSCI.0613-07.2007 17522311 PMC6672756

[B12] Chamberland S, Topolnik L (2012) Inhibitory control of hippocampal inhibitory neurons. Front Neurosci 6:165. 10.3389/fnins.2012.00165 23162426 PMC3496901

[B13] Chamero P, Marton TF, Logan DW, Flanagan K, Cruz JR, Saghatelian A, Cravatt BF, Stowers L (2007) Identification of protein pheromones that promote aggressive behaviour. Nature 450:899–902. 10.1038/nature05997 18064011

[B14] Chen TW, Wardill TJ, Sun Y, Pulver SR, Renninger SL, Baohan A, Schreiter ER, Kerr RA, Orger MB, Jayaraman V, Looger LL, Svoboda K, Kim DS (2013) Ultrasensitive fluorescent proteins for imaging neuronal activity. Nature 499:295–300. 10.1038/nature12354 23868258 PMC3777791

[B15] Chen WR, Xiong W, Shepherd GM (2000) Analysis of relations between NMDA receptors and GABA release at olfactory bulb reciprocal synapses. Neuron 25:625–633. 10.1016/s0896-6273(00)81065-x 10774730

[B16] Dana H, Sun Y, Mohar B, Hulse BK, Kerlin AM, Hasseman JP, Tsegaye G, Tsang A, Wong A, Patel R, Macklin JJ, Chen Y, Konnerth A, Jayaraman V, Looger LL, Schreiter ER, Svoboda K, Kim DS (2019) High-performance calcium sensors for imaging activity in neuronal populations and microcompartments. Nat Methods 16:649–657. 10.1038/s41592-019-0435-6 31209382

[B17] Doyle WI, Meeks JP (2017) Heterogeneous effects of noradrenaline on spontaneous and stimulus-driven activity in the male accessory olfactory bulb. J Neurophysiol 117:1342–1351. 10.1152/jn.00871.2016 28053247 PMC5350266

[B18] Doyle WI, Hammen GF, Meeks JP (2014) Ex vivo preparations of the intact vomeronasal organ and accessory olfactory bulb. J Vis Exp. Advance online publication. Retrieved Aug 4, 2014. doi: 10.3791/51813 10.3791/51813 PMC4299356 25145699

[B19] Doyle WI, Dinser JA, Cansler HL, Zhang X, Dinh DD, Browder NS, Riddington IM, Meeks JP (2016) Faecal bile acids are natural ligands of the mouse accessory olfactory system. Nat Commun 7:11936. 10.1038/ncomms11936 27324439 PMC4919516

[B20] Egger V, Svoboda K, Mainen ZF (2005) Dendrodendritic synaptic signals in olfactory bulb granule cells: local spine boost and global low-threshold spike. J Neurosci 25:3521–3530. 10.1523/JNEUROSCI.4746-04.2005 15814782 PMC6725376

[B21] Gao Y, Budlong C, Durlacher E, Davison IG (2017) Neural mechanisms of social learning in the female mouse. Elife 6:e25421 10.7554/eLife.25421 28621665 PMC5531829

[B22] Geramita M, Urban NN (2017) Differences in glomerular-layer-mediated feedforward inhibition onto mitral and tufted cells lead to distinct modes of intensity coding. J Neurosci 37:1428–1438. 10.1523/JNEUROSCI.2245-16.2016 28028200 PMC5299564

[B23] Gorin M, Tsitoura C, Kahan A, Watznauer K, Drose DR, Arts M, Mathar R, O'Connor S, Hanganu-Opatz IL, Ben-Shaul Y, Spehr M (2016) Interdependent conductances drive infraslow intrinsic rhythmogenesis in a subset of accessory olfactory bulb projection neurons. J Neurosci 36:3127–3144. 10.1523/JNEUROSCI.2520-15.2016 26985025 PMC6705527

[B24] Gutiérrez-Castellanos N, Pardo -Bellver C, Martínez -García F, Lanuza E (2014) The vomeronasal cortex—afferent and efferent projections of the posteromedial cortical nucleus of the amygdala in mice. Eur J Neurosci 39:141–158. 10.1111/ejn.12393 24188795

[B25] Haga S, Hattori T, Sato T, Sato K, Matsuda S, Kobayakawa R, Sakano H, Yoshihara Y, Kikusui T, Touhara K (2010) The male mouse pheromone ESP1 enhances female sexual receptive behaviour through a specific vomeronasal receptor. Nature 466:118–122. 10.1038/nature09142 20596023

[B26] Halabisky B, Friedman D, Radojicic M, Strowbridge BW (2000) Calcium influx through NMDA receptors directly evokes GABA release in olfactory bulb granule cells. J Neurosci 20:5124–5134. 10864969 10.1523/JNEUROSCI.20-13-05124.2000 PMC6772283

[B27] Hammen GF, Turaga D, Holy TE, Meeks JP (2014) Functional organization of glomerular maps in the mouse accessory olfactory bulb. Nat Neurosci 17:953–961. 10.1038/nn.3738 24880215 PMC4327767

[B28] Häusser M, Margrie TW (2014) Two-photon targeted patching and electroporation in vivo. Cold Spring Harb Protoc 2014:78–85. 10.1101/pdb.prot080143 24371321 PMC7616901

[B29] Hendrickson RC, Krauthamer S, Essenberg JM, Holy TE (2008) Inhibition shapes sex selectivity in the mouse accessory olfactory bulb. J Neurosci 28:12523–12534. 10.1523/JNEUROSCI.2715-08.2008 19020044 PMC6671729

[B30] Holy TE, Dulac C, Meister M (2000) Responses of vomeronasal neurons to natural stimuli. Science 289:1569–1572. 10.1126/science.289.5484.1569 10968796

[B31] Huang L, Garcia I, Jen H-I, Arenkiel BR (2013) Reciprocal connectivity between mitral cells and external plexiform layer interneurons in the mouse olfactory bulb. Front Neural Circuits 7:32. 10.3389/fncir.2013.00032 23459611 PMC3584718

[B32] Inoue M, Takeuchi A, Manita S, Horigane S-i, Sakamoto M, Kawakami R, Yamaguchi K, Otomo K, Yokoyama H, Kim R, Yokoyama T, Takemoto-Kimura S, Abe M, Okamura M, Kondo Y, Quirin S, Ramakrishnan C, Imamura T, Sakimura K, Nemoto T, et al (2019) Rational engineering of XCaMPs, a multicolor GECI suite for in vivo imaging of complex brain circuit dynamics. Cell 177:1346–1360.e24. 10.1016/j.cell.2019.04.007 31080068

[B33] Isaacson JS (2001) Mechanisms governing dendritic γ-aminobutyric acid (GABA) release in the rat olfactory bulb. Proc Natl Acad Sci USA 98:337–342. 10.1073/pnas.98.1.337 11120892 PMC14591

[B34] Isaacson JS, Strowbridge BW (1998) Olfactory reciprocal synapses: dendritic signaling in the CNS. Neuron 20:749–761. 10.1016/s0896-6273(00)81013-2 9581766

[B35] Jeanne JM, Wilson RI (2015) Convergence, divergence, and reconvergence in a feedforward network improves neural speed and accuracy. Neuron 88:1014–1026. 10.1016/j.neuron.2015.10.018 26586183 PMC5488793

[B36] Jia C, Chen WR, Shepherd GM (1999) Synaptic organization and neurotransmitters in the rat accessory olfactory bulb. J Neurophysiol 81:345–355. 10.1152/jn.1999.81.1.345 9914294

[B37] Kaba H, Keverne EB (1988) The effect of microinfusions of drugs into the accessory olfactory bulb on the olfactory block to pregnancy. Neuroscience 25:1007–1011. 10.1016/0306-4522(88)90053-x 2841623

[B38] Kaba H, Nakanishi S (1995) Synaptic mechanisms of olfactory recognition memory. Rev Neurosci 6:125–141. 10.1515/revneuro.1995.6.2.125 8564024

[B39] Kaba H, Huang G-Z (2005) Long-term potentiation in the accessory olfactory bulb: a mechanism for olfactory learning. Chem Senses 30 [Suppl 1]:i150–i151. 10.1093/chemse/bjh158 15738085

[B40] Kato HK, Gillet SN, Peters AJ, Isaacson JS, Komiyama T (2013) Parvalbumin-expressing interneurons linearly control olfactory bulb output. Neuron 80:1218–1231. 10.1016/j.neuron.2013.08.036 24239124 PMC3884945

[B41] Lage-Rupprecht V, Jodar T, Yeghiazaryan G, Rozsa B, Egger V (2018) Local reciprocal release of GABA from dendritic spines of olfactory bulb granule cells requires local sodium channel activation and occurs on both fast and slow timescales. bioRxiv 440198. doi: 10.1101/440198 10.1101/440198

[B42] Larriva-Sahd J (2008) The accessory olfactory bulb in the adult rat: a cytological study of its cell types, neuropil, neuronal modules, and interactions with the main olfactory system. J Comp Neurol 510:309–350. 10.1002/cne.21790 18634021

[B43] Liu G, Froudarakis E, Patel JM, Kochukov MY, Pekarek B, Hunt PJ, Patel M, Ung K, Fu C-H, Jo J, Lee H-K, Tolias AS, Arenkiel BR (2019) Target specific functions of EPL interneurons in olfactory circuits. Nat Commun 10:3369. 10.1038/s41467-019-11354-y 31358754 PMC6662826

[B44] Luo M, Fee MS, Katz LC (2003) Encoding pheromonal signals in the accessory olfactory bulb of behaving mice. Science 299:1196–1201. 10.1126/science.1082133 12595684

[B45] Madisen L, Zwingman TA, Sunkin SM, Oh SW, Zariwala HA, Gu H, Ng LL, Palmiter RD, Hawrylycz MJ, Jones AR, Lein ES, Zeng H (2010) A robust and high-throughput Cre reporting and characterization system for the whole mouse brain. Nat Neurosci 13:133–140. 10.1038/nn.2467 20023653 PMC2840225

[B46] Maksimova MA, Cansler HL, Zuk KE, Torres JM, Roberts DJ, Meeks JP (2019) Interneuron functional diversity in the mouse accessory olfactory bulb. eNeuro 6:ENEURO.0058-19.2019 10.1523/ENEURO.0058-19.2019 PMC6712203 31358509

[B47] Martinez-Marcos A (2009) On the organization of olfactory and vomeronasal cortices. Prog Neurobiol 87:21–30. 10.1016/j.pneurobio.2008.09.010 18929620

[B48] Matsuoka M, Kaba H, Mori Y, Neuroreport I-M (1997) Synaptic plasticity in olfactory memory formation in female mice. Neuroreport 8:2501–2504. 9261816 10.1097/00001756-199707280-00017

[B49] Matsuoka M, Kaba H, Moriya K, Yoshida-Matsuoka J, Costanzo RM, Norita M, Ichikawa M (2004) Remodeling of reciprocal synapses associated with persistence of long‐term memory. Eur J Neurosci 19:1668–1672. 10.1111/j.1460-9568.2004.03271.x 15066163

[B50] Meeks JP, Holy TE (2009) An ex vivo preparation of the intact mouse vomeronasal organ and accessory olfactory bulb. J Neurosci Methods 177:440–447. 10.1016/j.jneumeth.2008.11.013 19073215 PMC2709076

[B51] Meeks JP, Arnson HA, Holy TE (2010) Representation and transformation of sensory information in the mouse accessory olfactory system. Nat Neurosci 13:723–730. 10.1038/nn.2546 20453853 PMC2930753

[B52] Miyamichi K, Shlomai-Fuchs Y, Shu M, Weissbourd BC, Luo L, Mizrahi A (2013) Dissecting local circuits: parvalbumin interneurons underlie broad feedback control of olfactory bulb output. Neuron 80:1232–1245. 10.1016/j.neuron.2013.08.027 24239125 PMC3932159

[B53] Mohrhardt J, Nagel M, Fleck D, Ben-Shaul Y, Spehr M (2018) Signal Detection and Coding in the Accessory Olfactory System. Chem Senses 43:667–695. 10.1093/chemse/bjy061 30256909 PMC6211456

[B54] Moriya-Ito K, Endoh K, Fujiwara-Tsukamoto Y, Ichikawa M (2013) Three-dimensional reconstruction of electron micrographs reveals intrabulbar circuit differences between accessory and main olfactory bulbs. Front Neuroanat 7:5. 10.3389/fnana.2013.00005 23626525 PMC3631763

[B55] Nagai Y, Sano H, Yokoi M (2005) Transgenic expression of Cre recombinase in mitral/tufted cells of the olfactory bulb. Genesis 43:12–16. 10.1002/gene.20146 16106355

[B56] Nodari F, Hsu F-F, Fu X, Holekamp TF, Kao L-F, Turk J, Holy TE (2008) Sulfated steroids as natural ligands of mouse pheromone-sensing neurons. J Neurosci 28:6407–6418. 10.1523/JNEUROSCI.1425-08.2008 18562612 PMC2726112

[B57] Oboti L, Russo E, Tran T, Durstewitz D, Corbin JG (2018) Amygdala corticofugal input shapes mitral cell responses in the accessory olfactory bulb. eNeuro 5:ENEURO.0175-18.2018 10.1523/ENEURO.0175-18.2018 PMC6001136 29911171

[B58] Papes F, Logan DW, Stowers L (2010) The vomeronasal organ mediates interspecies defensive behaviors through detection of protein pheromone homologs. Cell 141:692–703. 10.1016/j.cell.2010.03.037 20478258 PMC2873972

[B59] Rothermel M, Brunert D, Zabawa C, Díaz-Quesada M, Wachowiak M (2013) Transgene expression in target-defined neuron populations mediated by retrograde infection with adeno-associated viral vectors. J Neurosci 33:15195–15206. 10.1523/JNEUROSCI.1618-13.2013 24048849 PMC3776063

[B60] Schoppa NE, Kinzie MJ, Sahara Y, Segerson TP, Westbrook GL (1998) Dendrodendritic inhibition in the olfactory bulb is driven by NMDA receptors. J Neurosci 18:6790–6802. 10.1523/JNEUROSCI.18-17-06790.1998 9712650 PMC6792983

[B61] Shipley MT, Adamek GD (1984) The connections of the mouse olfactory bulb: a study using orthograde and retrograde transport of wheat germ agglutinin conjugated to horseradish peroxidase. Brain Res Bull 12:669–688. 10.1016/0361-9230(84)90148-5 6206930

[B62] Smith RS, Hu R, DeSouza A, Eberly CL, Krahe K, Chan W, Araneda RC (2015) Differential muscarinic modulation in the olfactory bulb. J Neurosci 35:10773–10785. 10.1523/JNEUROSCI.0099-15.2015 26224860 PMC4518052

[B63] Stowers L, Liberles SD (2016) State-dependent responses to sex pheromones in mouse. Curr Opin Neurobiol 38:74–79. 10.1016/j.conb.2016.04.001 27093585 PMC4921285

[B64] Taniguchi H, He M, Wu P, Kim S, Paik R, Sugino K, Kvitsiani D, Kvitsani D, Fu Y, Lu J, Lin Y, Miyoshi G, Shima Y, Fishell G, Nelson SB, Huang ZJ (2011) A resource of Cre driver lines for genetic targeting of GABAergic neurons in cerebral cortex. Neuron 71:995–1013. 10.1016/j.neuron.2011.07.026 21943598 PMC3779648

[B65] Taniguchi M, Kaba H (2001) Properties of reciprocal synapses in the mouse accessory olfactory bulb. Neuroscience 108:365–370. 10.1016/s0306-4522(01)00427-4 11738251

[B66] Tolokh II, Fu X, Holy TE (2013) Reliable sex and strain discrimination in the mouse vomeronasal organ and accessory olfactory bulb. J Neurosci 33:13903–13913. 10.1523/JNEUROSCI.0037-13.2013 23966710 PMC3755725

[B67] Turaga D, Holy TE (2012) Organization of vomeronasal sensory coding revealed by fast volumetric calcium imaging. J Neurosci 32:1612–1621. 10.1523/JNEUROSCI.5339-11.2012 22302803 PMC3342671

[B68] Wagner S, Gresser AL, Torello AT, Dulac C (2006) A multireceptor genetic approach uncovers an ordered integration of VNO sensory inputs in the accessory olfactory bulb. Neuron 50:697–709. 10.1016/j.neuron.2006.04.033 16731509

[B69] Wei Z, Lin B-J, Chen T-W, Daie K, Svoboda K, Druckmann S (2019) A comparison of neuronal population dynamics measured with calcium imaging and electrophysiology. bioRxiv 840686. doi: 10.1101/840686 10.1101/840686 PMC7518847 32931495

[B70] Wilson NR, Runyan CA, Wang FL, Sur M (2012) Division and subtraction by distinct cortical inhibitory networks in vivo. Nature 488:343–348. 10.1038/nature11347 22878717 PMC3653570

[B71] Yoles-Frenkel M, Kahan A, Ben-Shaul Y (2018) Temporal response properties of accessory olfactory bulb neurons: limitations and opportunities for decoding. J Neurosci 38:4957–4976. 10.1523/JNEUROSCI.2091-17.2018 29712784 PMC6596120

[B72] Zhu P, Frank T, Friedrich RW (2013) Equalization of odor representations by a network of electrically coupled inhibitory interneurons. Nat Neurosci 16:1678–1686. 10.1038/nn.3528 24077563

